# A bacterial ABC transporter enables import of mammalian host glycosaminoglycans

**DOI:** 10.1038/s41598-017-00917-y

**Published:** 2017-04-21

**Authors:** Sayoko Oiki, Bunzo Mikami, Yukie Maruyama, Kousaku Murata, Wataru Hashimoto

**Affiliations:** 1 Laboratory of Basic and Applied Molecular Biotechnology, Division of Food Science and Biotechnology, Graduate School of Agriculture, Kyoto University, Uji, Kyoto 611-0011 Japan; 2 Laboratory of Applied Structural Biology, Division of Applied Life Sciences, Graduate School of Agriculture, Kyoto University, Uji, Kyoto 611-0011 Japan; 3 Laboratory of Food Microbiology, Department of Life Science, Faculty of Science and Engineering, Setsunan University, Neyagawa, Osaka 572-8508 Japan

## Abstract

Glycosaminoglycans (GAGs), such as hyaluronan, chondroitin sulfate, and heparin, constitute mammalian extracellular matrices. The uronate and amino sugar residues in hyaluronan and chondroitin sulfate are linked by 1,3-glycoside bond, while heparin contains 1,4-glycoside bond. Some bacteria target GAGs as means of establishing colonization and/or infection, and bacterial degradation mechanisms of GAGs have been well characterized. However, little is known about the bacterial import of GAGs. Here, we show a GAG import system, comprised of a solute-binding protein (Smon0123)-dependent ATP-binding cassette (ABC) transporter, in the pathogenic *Streptobacillus moniliformis*. A genetic cluster responsible for depolymerization, degradation, and metabolism of GAGs as well as the ABC transporter system was found in the *S. moniliformis* genome. This bacterium degraded hyaluronan and chondroitin sulfate with an expression of the genetic cluster, while heparin repressed the bacterial growth. The purified recombinant Smon0123 exhibited an affinity with disaccharides generated from hyaluronan and chondroitin sulfate. X-ray crystallography indicated binding mode of Smon0123 to GAG disaccharides. The purified recombinant ABC transporter as a tetramer (Smon0121-Smon0122/Smon0120-Smon0120) reconstructed in liposomes enhanced its ATPase activity in the presence of Smon0123 and GAG disaccharides. This is the first report that has molecularly depicted a bacterial import system of both sulfated and non-sulfated GAGs.

## Introduction

Extracellular matrices are ubiquitously present in all mammalian tissues and organs and serve as physical scaffolds for cellular constituents, cell differentiation, homeostasis, and tissue formation^1^. Glycosaminoglycans (GAGs), major components of extracellular matrices, are acidic heteropolysaccharides with a typical disaccharide (uronate and amino sugar)-repeating unit^2^. GAGs are classified as hyaluronan, chondroitin sulfate, heparin, or heparan sulfate based on constituent sugar, mode of glycoside bond, and sulfation level^3^. Hyaluronan consists of d-glucuronic acid (GlcUA) and *N*-acetyl-d-glucosamine (GlcNAc), and chondroitin sulfate is comprised of GlcUA and *N*-acetyl-d-galactosamine (GalNAc) with a sulfate group(s) at position 4 or 6 or both. Heparin and heparan sulfate are composed of GlcUA or l-iduronic acid (IdoUA) and d-glucosamine (GlcN) or GlcNAc^4^. Residues, uronate and amino sugar, in hyaluronan and chondroitin sulfate are linked by 1,3-glycoside bonds, while residues in heparin and heparan sulfate are linked by 1,4-glycoside bonds. With the exception of hyaluronan, these GAGs frequently contain sulfate groups in uronate and/or amino sugar residues.

**Figure 1:**
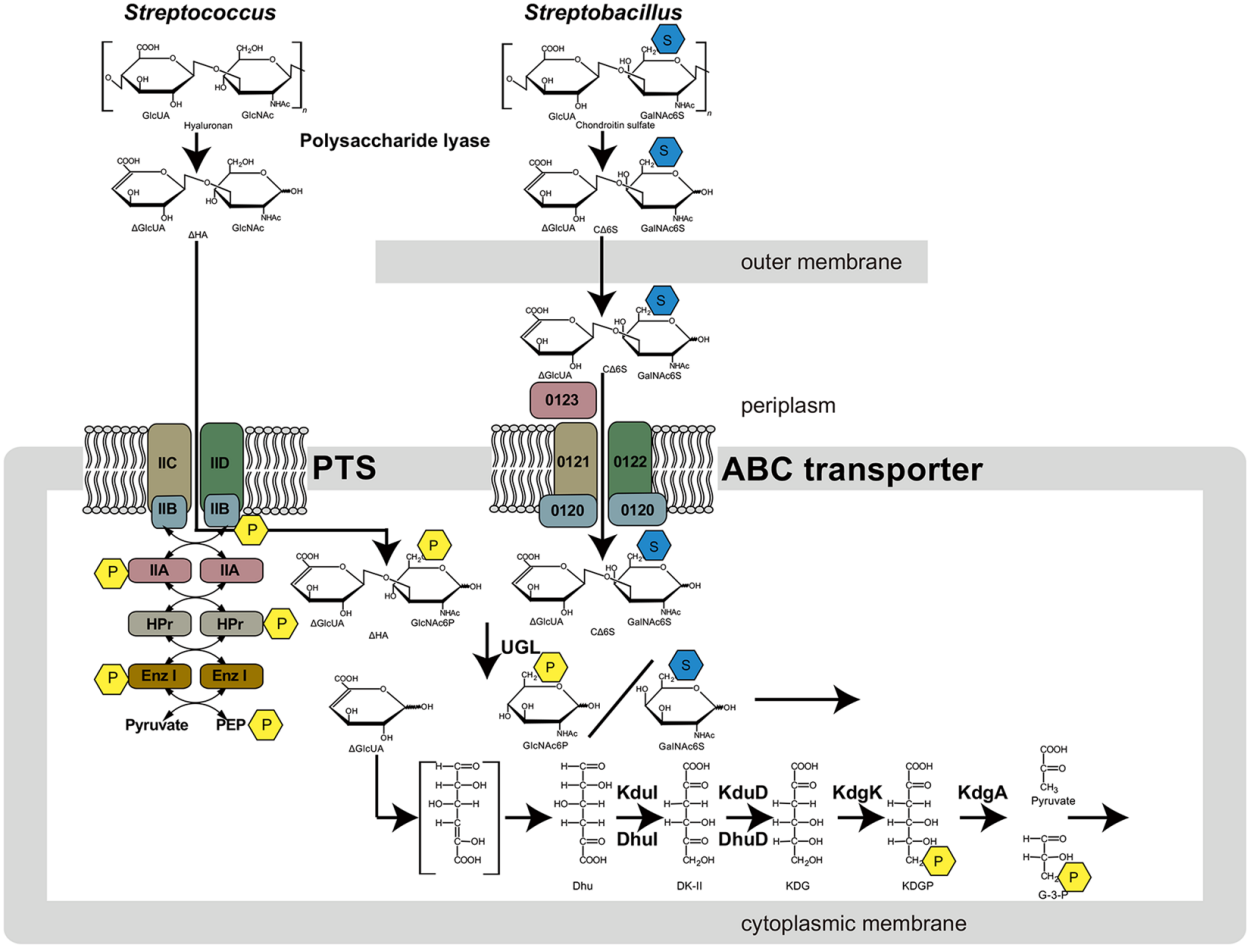
Bacterial system for degradation and import of GAGs. GAGs such as hyaluronan and chondroitin sulfate are depolymerized to unsaturated disaccharides by extracellular or cell-surface polysaccharide lyases. The resultant unsaturated disaccharides are incorporated to cytoplasm by PTS or periplasmic binding protein-dependent ABC transporter. In the case of PTS, substrates (unsaturated disaccharides) are phosphorylated across the cytoplasmic membrane. Unsaturated disaccharides are degraded to constituent monosaccharides (unsaturated uronate and amino sugar) by UGL, and the resultant unsaturated uronate is metabolized to pyruvate and glyceraldehyde-3-phosphate (G-3-P) by subsequent reactions of isomerase, reductase, kinase and aldolase. The pathway and proteins/enzymes are detailed in the text. P enclosed in a yellow hexagon, phosphate group; S enclosed in a cyan hexagon, sulfate group.

**Figure 2:**
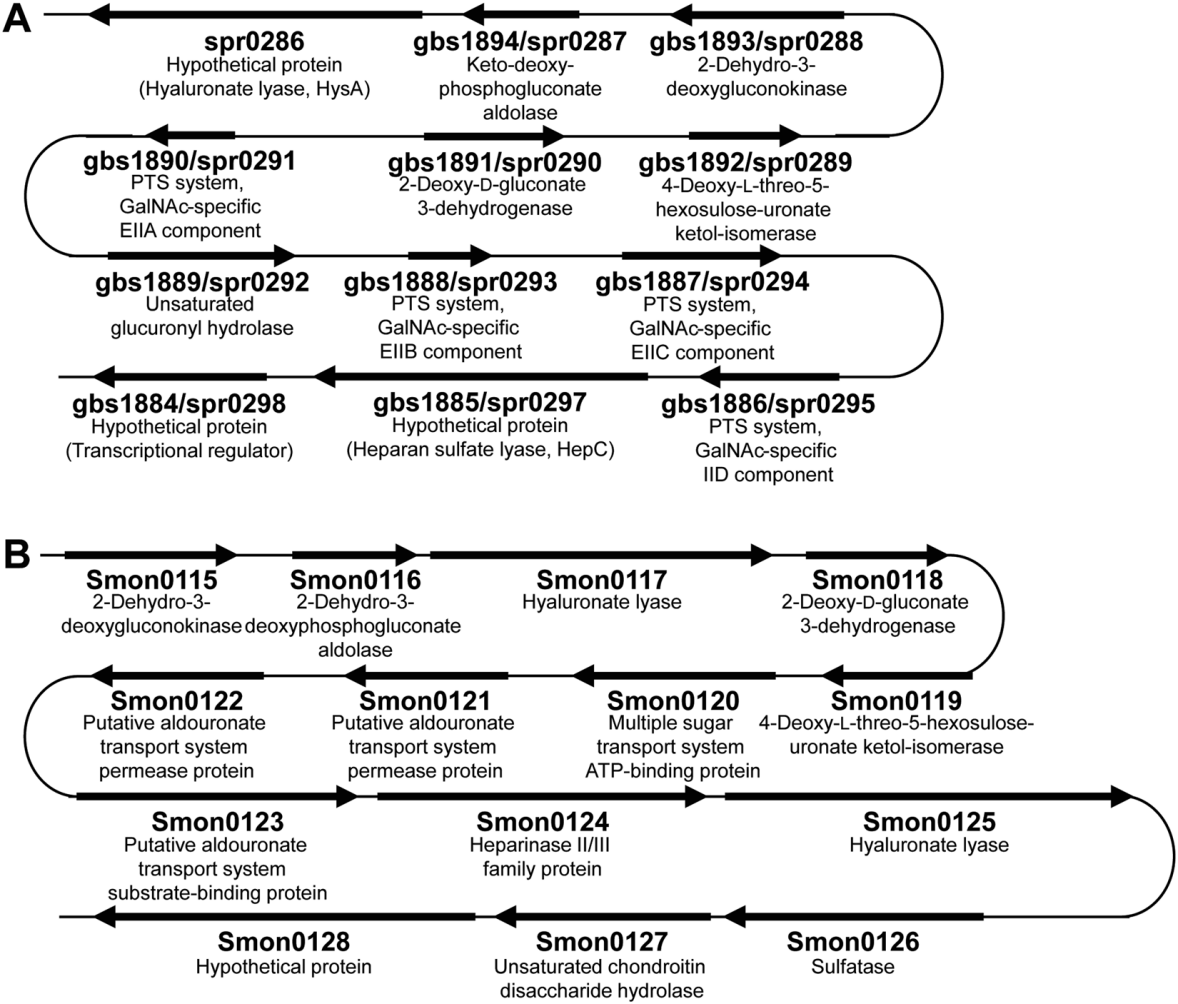
Bacterial GAG genetic clusters. (**A**) Genetic cluster of *Streptococcus agalactiae*/*Streptococcus pneumoniae*. (**B**) Genetic cluster of *Streptobacillus moniliformis*. Genes for GAG depolymerization, degradation, and metabolism, except for import, are well conserved between the two bacterial clusters. Genetic clusters of *Streptococcus* and *Streptobacillus* encode PTS and the solute-binding protein-dependent ABC transporter, respectively, as an importer.

Some pathogenic bacteria target mammalian GAGs as a means of establishing colonization and/or infection^5^. Particularly, in pathogenic Gram-positive bacteria, such as staphylococci and streptococci, GAGs (e.g., hyaluronan and chondroitin sulfate) are depolymerized to unsaturated disaccharides by extracellular or cell-surface polysaccharide lyase (HysA) through a β-elimination reaction^6–8^ (Fig. 1). Our previous reports indicated that resultant unsaturated GAG disaccharides with C=C double bonds at non-reducing termini are degraded to constituent monosaccharides (i.e., unsaturated uronate and amino sugar) by unsaturated glucuronyl hydrolase (UGL) through hydration of C=C double bonds in the cytoplasm^9, 10^. More recently, it was demonstrated that unsaturated uronate is metabolized to pyruvate and glyceraldehyde-3-phosphate by subsequent reactions of isomerase (DhuI), NADH-dependent reductase (DhuD), kinase (KdgK), and aldolase (KdgA)^11^. In the streptococcal genome, these enzymes (HysA, UGL, DhuI, DhuD, KdgK, and KdgA) are encoded as a GAG genetic cluster, which is crucial for depolymerization, degradation, and metabolism of GAGs (Fig. 2A). A putative phosphotransferase system (PTS), also part of the streptococcal GAG genetic cluster, is expressed in the presence of hyaluronan^12^, and mutated PTS genes resulted in failed utilization of hyaluronan^13^, suggesting that the PTS is involved in bacterial import of the non-sulfated GAG.

To note, PTS, a sugar transport system specific to bacteria, is composed of Enzyme I (EI), histidine-containing phosphocarrier protein (HPr), and Enzyme II (EII) with hetero-subunits (e.g., IIA, IIB, IIC, and IID)^14^. EI and HPr are located in the cytoplasm and non-specifically recognize PTS substrates; EII, which consists of cell membrane and cytoplasmic domains, specifically recognizes PTS substrates. Mechanistically, PTS mediates sugar uptake via phosphorylating sugar substrates at the C-6 position through an initial phosphotransfer from phosphoenolpyruvate (PEP) and involvement of EI, HPr, and EII^15^. Because a large number of GAGs, except for hyaluronan, are frequently sulfated at the C-6 position^16^, unsaturated GAG disaccharides sulfated at the C-6 position may be unsuitable PTS substrates. In fact, more than 20 sugars have been identified to be imported by PTS, though no sugars with a modification at C-6 position are included in these substrates^17^.

In addition, the pathogenic Gram-negative bacterium *Streptobacillus moniliformis* has a similar GAG genetic cluster (Fig. 2B). *S. moniliformis* usually colonizes the rodent oral cavity and is the causative agent of Rat-bite fever in humans either directly or indirectly exposed to rodents^18^. Rat-bite fever is characterized by polyarthralgia, high fever, and headache, and its fatality rate reaches to 10% among untreated patients^19^. In the *Streptobacillus* GAG genetic cluster, a periplasmic solute-binding protein-dependent ATP-binding cassette (ABC) transporter is encoded in place of PTS. Because ABC transporters incorporate substrates into the cytoplasm without any modifications^20^, we hypothesized that GAGs sulfated at C-6 position should be viable substrates for ABC transporters, which is confirmed by this study.

In addition to bacterial depolymerization and degradation of GAGs, investigating the GAG import system is important to further elucidate bacterial physiology and pathogenicity. For some bacteria among normal bacterial flora, the GAG import system is required for assimilation of GAG present on host cells. Generally, bacterial polysaccharide-degrading enzymes are expressed under the control of cytoplasmic transcriptional regulators that sense substrates^21, 22^. Inhibition of the import system results in reduced expression of these degrading enzymes, which may be a means to minimize the brunt of bacterial GAG-dependent infectious diseases. This study aimed to identify molecular components of the *S. moniliformis* GAG import system, structurally determine the GAG import proteins that interact and bind to sulfated and non-sulfated GAG disaccharides via X-ray crystallography, and functionally characterize the ABC transporter.

## Results

### *Streptobacillus moniliformis* GAG genetic cluster

Our previous reports^11, 12^ indicated the presence of a genetic cluster responsible for depolymerization, degradation, and metabolism of GAGs in pathogenic bacteria, such as streptococci, enterococci, and clostridia. These bacterial GAG genetic clusters encode polysaccharide lyase and UGL. Hyaluronate and heparan sulfate lyases encoded by the cluster are classified in polysaccharide lyase (PL) families 8 and 12, respectively, as per the Carbohydrate Active enZyme (CAZy) database^23^, while all UGLs are solely members of the glycoside hydrolase (GH) family 88 according to the database. These depolymerizing and degrading enzymes are well conserved among bacterial GAG genetic clusters. In addition to such enzymes, genes coding for PTS are frequently observed in the cluster. There are, however, some variations in constitutive genes among GAG-related bacteria. For example, isomerases (streptococcal DhuI and clostridial KduI) and reductases (streptococcal DhuD and clostridial KduD) function as 4-deoxy-L-*threo*-5-hexosulose-uronate ketol-isomerase and 2-keto-3-deoxy-D-gluconate dehydrogenase, respectively, although little homology is observed between DhuI and KduI and between DhuD and KduD^11^. Thus, we decided to investigate GAG cluster organization in genome-sequenced bacteria.

Interestingly, the Gram-negative bacterium *S. moniliformis* ^24^ possessed a similar GAG genetic cluster containing genes which encode for a solute-binding protein-dependent ABC transporter (Fig. 2B). Based on the primary structure, the proteins encoded in the genetic cluster were annotated as follows: Smon0115, 2-dehydro-3-deoxygluconokinase (KdgK); Smon0116, 2-dehydro-3-deoxyphosphogluconate aldolase (KdgA); Smon0117, PL family 8 hyaluronate lyase (HysA); Smon0118, 2-deoxy-D-gluconate 3-dehydrogenase (KduD); Smon0119, 4-deoxy-L-*threo*-5-hexosulose-uronate ketol-isomerase (KduI); Smon0120, multiple sugar transport system (ATP-binding protein); Smon0121, putative aldouronate transport system (permease protein); Smon0122, putative aldouronate transport system (permease protein); Smon0123, putative aldouronate transport system (substrate-binding protein); Smon0124, PL family 12 heparinase II/III family protein (HepC); Smon0125, PL family 8 hyaluronate lyase (HysA); Smon0126, sulfatase; Smon0127, GH family 88 unsaturated chondroitin disaccharide hydrolase (UGL); Smon0128, hypothetical protein.

Some streptococci can assimilate hyaluronan as a sole carbon source^25, 26^, and PTS encoded in the streptococcal GAG genetic cluster is suitable for import of unsaturated hyaluronan disaccharides containing no sulfate groups (Fig. 1, left). On the other hand, unsaturated disaccharides with a sulfate group at the C-6 position generated from sulfated GAGs, e.g., chondroitin sulfate C, are unsuitable for PTS substrates. The ABC transporter possibly enables bacteria to incorporate sulfated disaccharides. Thus, degradation and import systems of GAGs were analyzed in *S. moniliformis*.

### *S. moniliformis* degrades GAGs

**Figure 3:**
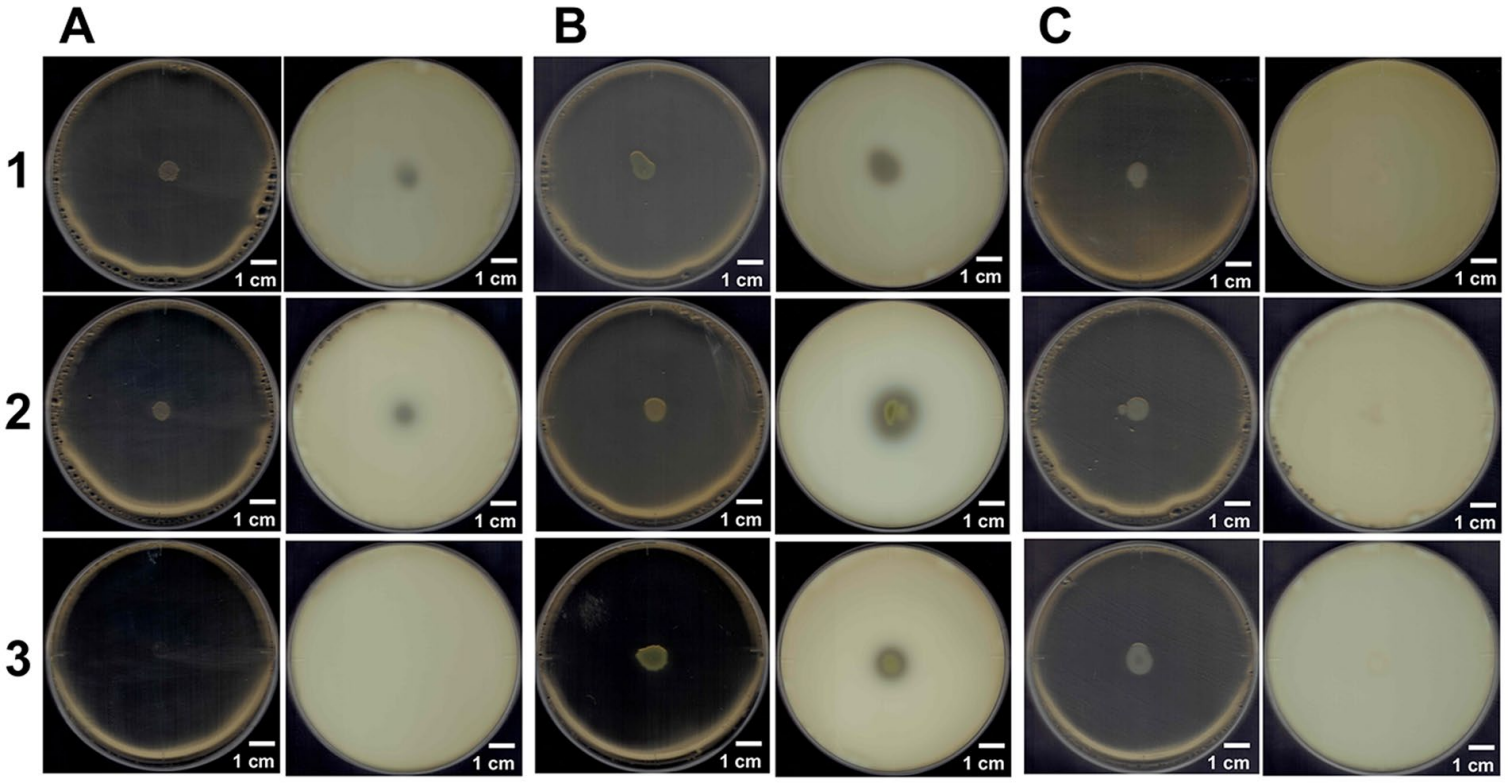
Degradation of GAGs. (**A**) *S. moniliformis*. (**B**) *P. heparinus* as a positive control. (**C**) *E. coli* as a negative control. Plates on the left and right represent before and after addition of acetic acid, respectively. Plates 1, 2, and 3 contained hyaluronan, chondroitin sulfate C, and heparin, respectively.

To investigate degradation of GAGs by *S. moniliformis*, a plate method for halo detection^27^ was adopted (Fig. 3). Bacterial cells were grown on nutrient medium plates containing bovine serum albumin (BSA) and each of the GAGs, and acetic acid was poured on the plates after colony formation. Degradation of GAGs caused clear zones, halos, although GAGs resulted in formation of white precipitates due to interaction with BSA in the presence of acetic acid. Hyaluronan, chondroitin sulfate C, and heparin were used as substrates. Chondroitin sulfate is classified as chondroitin sulfates A, B, and C based on position of the sulfate group^28^. Chondroitin sulfate C is sulfated at the C-6 position of GalNAc, while chondroitin sulfates A and B are sulfated at the C-4 position of GalNAc. The repeating units of chondroitin sulfates A, B, and C are GlcUA-GalNAc4S (GalNAc with a sulfate group at C-4 position), IdoUA-GalNAc4S, and GlcUA-GalNAc6S (GalNAc with a sulfate group at C-6 position), respectively^29^. Gram-negative *Pedobacter heparinus* is known to degrade hyaluronan, chondroitin sulfate, and heparin^30^ and served as a positive control, while *Escherichia coli* served as a negative control.

*P. heparinus* cells formed halos on each GAG plate after addition of acetic acid (Fig. 3B; plates 1, 2, and 3 on the right), while no halo formation was observed on *E. coli* plates (Fig. 3C; plates 1, 2, and 3 on the right). In the case of *S. moniliformis*, halos were observed on plates containing hyaluronan or chondroitin sulfate C (Fig. 3A; plates 1 and 2, right). Surprisingly, the bacterium did not grow in the presence of heparin (Fig. 3A; plate 3, left). *S. moniliformis* in the presence of hyaluronan and chondroitin sulfate C in the nutrient medium grew better than when in the absence of GAG (Fig. S1).

### Expression of the GAG genetic cluster in *S. moniliformis*

**Figure 4:**
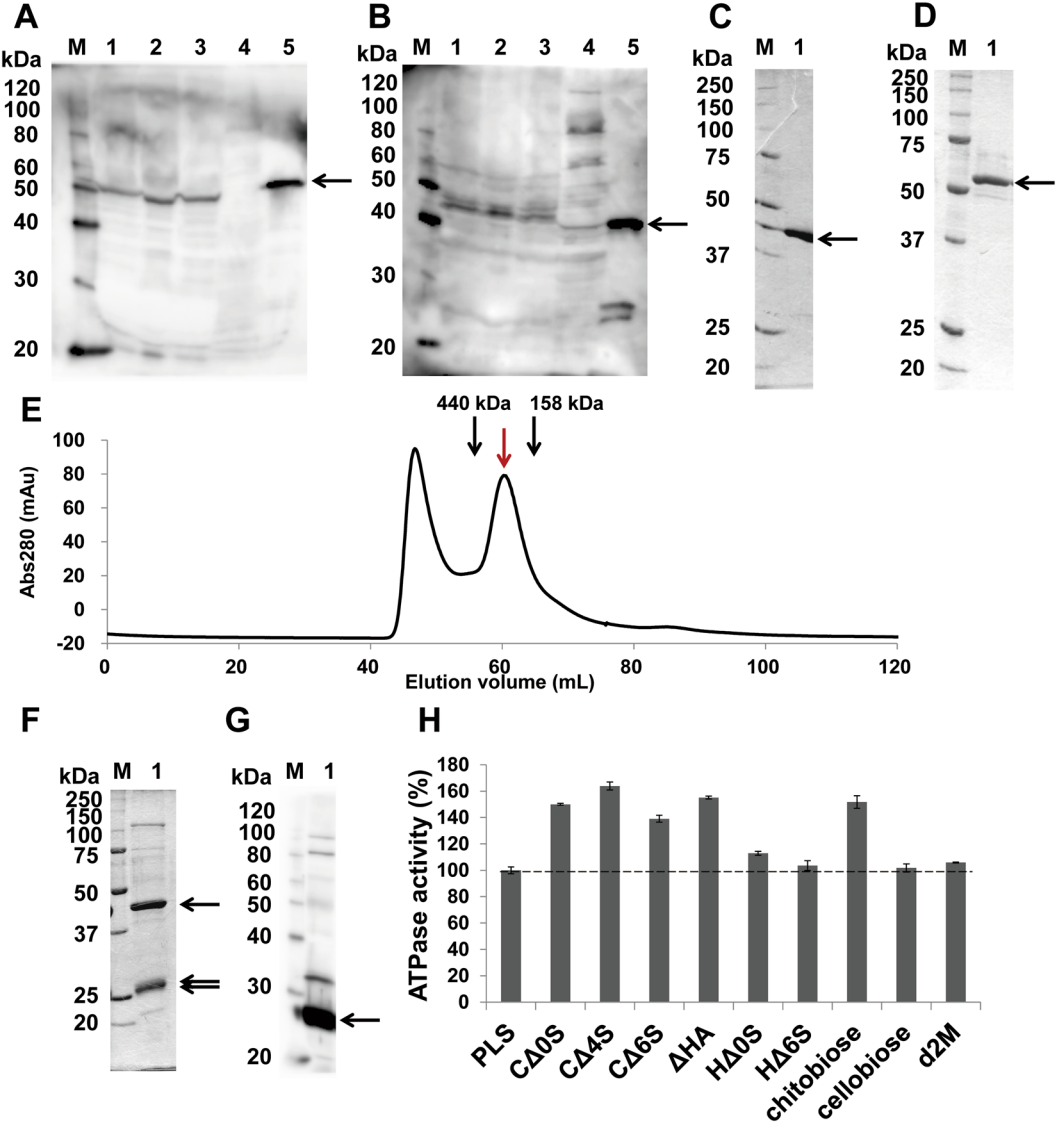
Expression and characterization of binding protein-dependent ABC transporter. SDS-PAGE followed by immunoblotting using anti-Smon0123 antibodies (**A**) and anti-SpyUGL antibodies (**B**). *S. moniliformis* cells grown in liquid nutrient medium (100 μl) in the presence or absence of GAG (hyaluronan or chondroitin sulfate (**C**) were collected at OD_600_ = 0.37 by centrifugation. The cells were lysed with SDS, and the resultant cell lysates were subjected to immunoblotting for detection of Smon0123 (solute-binding protein) and Smon0127 (UGL) encoded in the GAG genetic cluster. Lane M, protein standards with molecular masses of 120, 100, 80, 60, 50, 40, 30, and 20 kDa used for immunoblotting; lane 1, *S. moniliformis* cells in the absence of GAG; lane 2, *S. moniliformis* cells in the presence of hyaluronan; lane 3, *S. moniliformis* cells in the presence of chondroitin sulfate C; lane 4, *E. coli* cells in the absence of GAG; lane 5, positive control [purified Smon0123 (**A**) and SagUGL (**B**)]. (**C**) and (**D**) SDS-PAGE followed by staining with Coomassie Brilliant Blue (CBB). Lane M, protein standards with molecular masses of 250, 150, 100, 75, 50, 37, 25, and 20 kDa for CBB staining; lane 1, purified Smon0127 (**C**) and Smon0123 (**D**). (**E**) Elution profile of Smon0121-Smon0122(10xHis)/Smon0120-Smon0120 via gel filtration chromatography. Left and right-sided peaks show an aggregate and a tetramer, respectively. Volumes required for elution of the standard ferritin (440 kDa) and aldolase (158 kDa) are indicated by black arrows. (**F**) SDS-PAGE followed by CBB staining. Lane M, protein standards with molecular masses of 250, 150, 100, 75, 50, 37, 25, and 20 kDa; lane 1, purified Smon0121-Smon0122(10xHis)/Smon0120-Smon0120. (**G**) Immunoblotting using anti-histidine tag antibodies. Lane M, protein standards with molecular masses of 120, 100, 80, 60, 50, 40, 30, and 20 kDa; lane 1, purified Smon0121-Smon0122(10xHis)/Smon0120-Smon0120. (**H**) ATPase activity of the Smon0121-Smon0122(10xHis)/Smon0120-Smon0120 in liposomes in the presence or absence of various disaccharides. PLS, proteoliposome without disaccharides; chitobiose, *N*,*N*′-diacetylchitobiose; d2M, unsaturated alginate disaccharide. The ATPase activity in PLS was considered 100%. Each data represents the average of triplicate individual experiments (means ± standard errors of the means).

Because *S. moniliformis* degrades hyaluronan and chondroitin sulfate C, the expression of the bacterial GAG genetic cluster was examined by immunoblot analysis. The molecular masses of Smon0123 and Smon0127 were estimated to be 57 and 46 kDa, respectively, although Smon0123 was predicted to contain a signal peptide with a molecular mass of 2 kDa by a LipoP program^31^. As described later, anti-Smon0123 antibodies were prepared using recombinant Smon0123 as antigens. As Smon0127 exhibits a significant sequence identity (48%) with *Streptococcus pyogenes* UGL (SpyUGL), anti-SpyUGL antibodies^12^ were used to detect Smon0127. The purified recombinant Smon0123 and *Streptococcus agalactiae* UGL (SagUGL)^32^ were subjected to immunoblotting as the positive control (Fig. 4A and B, lane 5). Both Smon0123 and Smon0127 were constitutively expressed in bacterial cells grown in the GAG-free medium (Fig. 4A and B, lane 1). The intensity of both protein bands increased in the presence of GAG, particularly hyaluronan (Fig. 4A and B, lane 2). These results indicated that the GAG genetic cluster was constitutively expressed in *S. moniliformis* cells and that its expression was enhanced in the presence of GAG.

### Enzymatic properties of Smon0127

To investigate the enzymatic properties of Smon0127 expressed in bacterial cells, the overexpression system of Smon0127 was constructed in *E. coli* cells, and recombinant Smon0127 was expressed and purified to homogeneity (Fig. 4C). Absorbance at 235 nm derived from the C=C double bond in unsaturated hyaluronan disaccharide (ΔHA) decreased by the addition of Smon0127 to the substrate, indicating that Smon0127 functions as UGL. To determine the substrate specificity of Smon0127 UGL, various unsaturated GAG disaccharides, such as ΔHA, unsaturated chondroitin disaccharides (CΔ0S, CΔ4S, and CΔ6S), and unsaturated heparin disaccharides (HΔ0S and HΔ6S), were used as substrates. The difference in each substrate is as follows: CΔ0S, sulfate group-free unsaturated chondroitin disaccharide; CΔ4S, unsaturated chondroitin disaccharide with a sulfate group at the C-4 position of GalNAc; CΔ6S, unsaturated chondroitin disaccharide with a sulfate group at the C-6 position of GalNAc; HΔ0S, sulfate group-free unsaturated heparin disaccharide; and HΔ6S, unsaturated heparin disaccharide with a sulfate group at the C-6 position of GlcNAc.

Smon0127 UGL was the most active on CΔ0S (specific activity, 4.46 units/mg). Specific activities of the enzyme toward each substrate were as follows: ΔHA, 2.26 units/mg; CΔ4S, 0.342 units/mg; CΔ6S, 0.272 units/mg; HΔ0S, 0.0441 units/mg; and HΔ6S 0.0436 units/mg. The kinetic parameters of Smon0127 UGL are shown in the Supplementary Information.

### Interaction between Smon0123 and unsaturated GAG disaccharides

**Figure 5:**
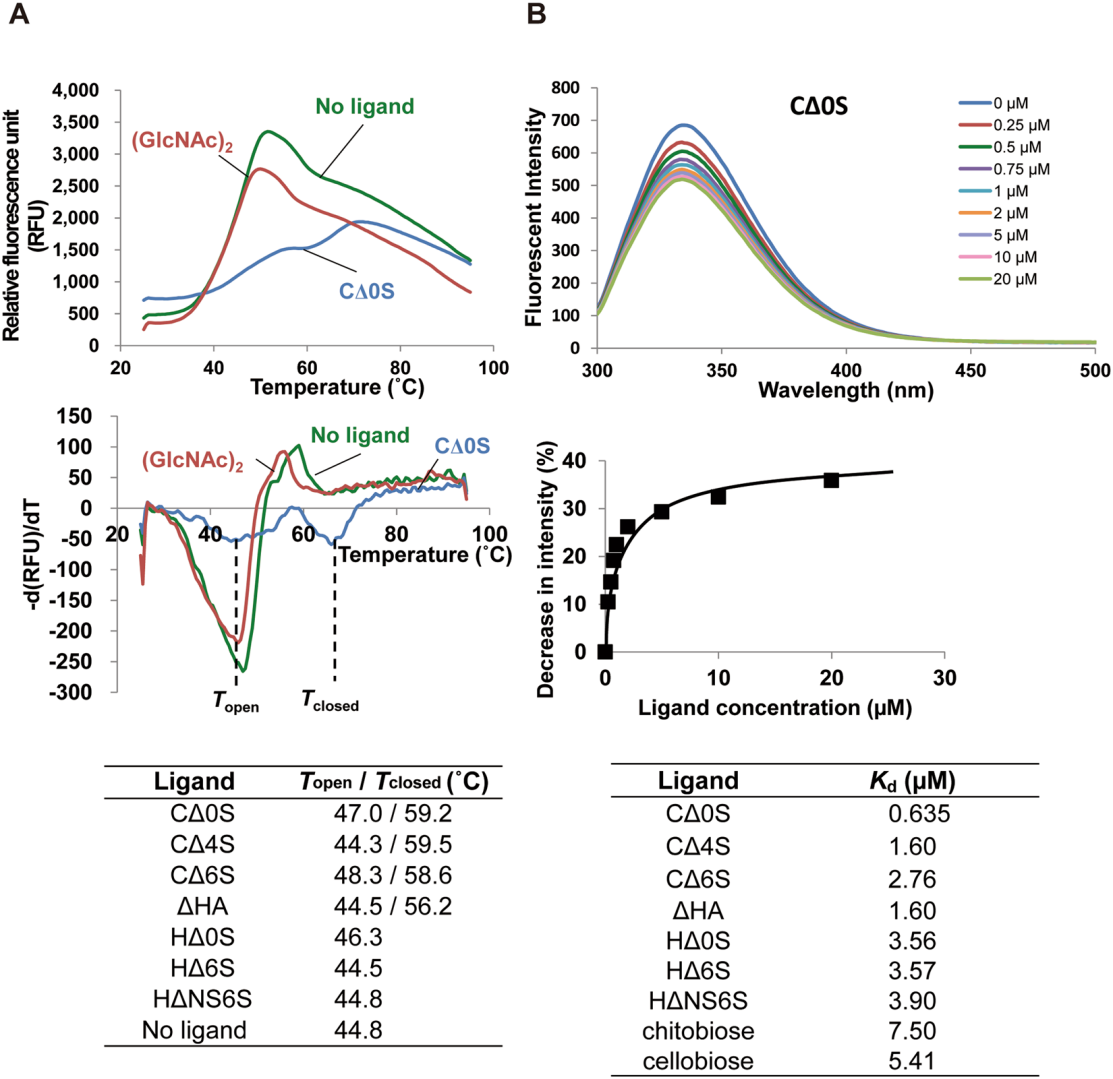
Affinity of Smon0123 with unsaturated GAG disaccharides. (**A**) DSF analysis. Upper, fluorescence profile of Smon0123 with C∆0S (green: 0 mM and cyan: 1 mM) or 1 mM *N*,*N*′-diacetylchitobiose (red). Middle, negative derivative curve plot derived from the fluorescence profile. Lower, the values of *T*_open_ and *T*_closed_ in the presence or absence of various unsaturated GAG disaccharides. (**B**) Fluorescence spectrum analysis. Upper, wavelength-scanned fluorescence intensity of Smon0123 with C∆0S (blue: 0 μM; red: 0.25 μM; green: 0.50 μM; purple: 0.75 μM; cyan: 1.0 μM; orange: 2.0 μM; lilac: 5.0 μM; pink: 10 μM; and olive: 20 μM). Middle, the relative fluorescence intensity by addition of increasing ligand concentrations was plotted after modification based on volume change in the cuvette. *K*_d_ was determined using the least-squares method. Lower, dissociation constants of Smon0123 with various unsaturated GAG disaccharides. Chitobiose, *N*,*N*′-diacetylchitobiose.

Recombinant Smon0123, the putative solute-binding protein, was expressed in *E. coli* cells, purified to homogeneity (Fig. 4D), and subjected to differential scanning fluorimetry (DSF)^33^ to examine the interaction between Smon0123 and unsaturated GAG disaccharides (Fig. 5A). Our previous report evidenced that this method is feasible for analyzing the interaction between the solute-binding protein and the substrate^34^. In DSF, the reaction temperature was escalated in the mixture of a protein, ligand, and fluorescent chemical (SYPRO Orange). The relative fluorescent unit (RFU) was changed due to the formation of hydrophobic interaction between unfolded protein and SYPRO Orange. The inflection point of the increase on the RFU is defined as the melting temperature (*T*_m_)^33^. As the ligand-bound protein is thermally more stable than the ligand-free form due to the conversion of the rigid form, *T*_m_ in the presence of a ligand was higher than that in the absence of a ligand. Generally, the solute-binding protein adopts two conformations during substrate binding, substrate-free open form and substrate-bound closed form^35^, indicating that there is a transitional state (substrate-bound open form) in the conformational change. This conformational change due to the interaction between the protein and the ligand is represented as two *T*_m_ values, *T*_open_ and *T*_closed_, corresponding to the substrate-bound open form and substrate-bound closed form, respectively.

Smon0123 in the presence of CΔ0S at 1 mM gave two *T*_m_ values (44.4 and 66.0 °C), while Smon0123 in the absence of a ligand gave a single *T*_m_ value (46.7 °C). *T*_closed_ (66.0 °C) was significantly higher than *T*_m_ (46.7 °C) (Fig. 5A, middle), suggesting that Smon0123 had a significant affinity with CΔ0S through the conformational change. On the other hand, the fluorescence profile of Smon0123 in the presence of the non-GAG disaccharide, *N*,*N*′-diacetylchitobiose (single *T*_m_, 45.6 °C) at 1 mM was comparable with that in the absence of ligand. Various unsaturated GAG disaccharides were used at 50 µM in a similar manner (Fig. 5A, lower). Two *T*_m_ values (*T*_open_ and *T*_closed_) of Smon0123 in the presence of CΔ0S, CΔ4S, CΔ6S, or ΔHA were detected, while each single *T*_m_ value of Smon0123 in the presence of HΔ0S, HΔ6S, or HΔNS6S (unsaturated heparin disaccharide with two sulfate groups at the N position and C-6 position of GlcNAc) kept low at around 45 °C. These data suggest that Smon0123 binds to unsaturated GAG disaccharides with the 1,3-glycoside bond (CΔ0S, CΔ4S, CΔ6S, and ΔHA) rather than the 1,4 bond (HΔ0S, HΔ6S, and HΔNS6S).

Due to the difficulty in determining the affinity level by DSF, interactions between Smon0123 and unsaturated GAG disaccharides were further analyzed by measuring the fluorescence intensity of Smon0123 in the presence or absence of ligands (Fig. 5B). The fluorescence intensity derived from tryptophan residues of Smon0123 proportionally decreased to increasing ligand concentrations (Fig. 5B, upper). The ratio of decreasing fluorescence intensity was plotted with respect to ligand concentration, and the dissociation constants were determined from the saturation curve (Fig. 5B, middle). Smon0123 showed the highest affinity with CΔ0S (*K*_d_, 0.635 ± 0.122 µM), followed by ΔHA, CΔ4S, and CΔ6S (Fig. 5B, lower). The affinity level of Smon0123 with unsaturated heparin disaccharides was low. Although *K*_d_ values for *N*,*N*′-diacetylchitobiose (7.50 ± 1.26 µM) or cellobiose (5.41 ± 0.606 µM) were also calculated, these were estimated to basal levels in this assay. The binding ability of Smon0123 to GAG polysaccharides (hyaluronan, chondroitin sulfate C, and heparin) was also investigated and no specific interaction between Smon0123 and these GAG polysaccharides was observed.

### Crystal structure of Smon0123

Because Smon0123 exhibited an affinity with sulfate-free and -bound unsaturated chondroitin disaccharides (CΔ0S, CΔ4S, and CΔ6S), the binding mode of Smon0123 to these substrates was analyzed by X-ray crystallography. The detailed results regarding crystallization and structure determination of Smon0123 are shown in the Supplementary Information.

**Figure 6:**
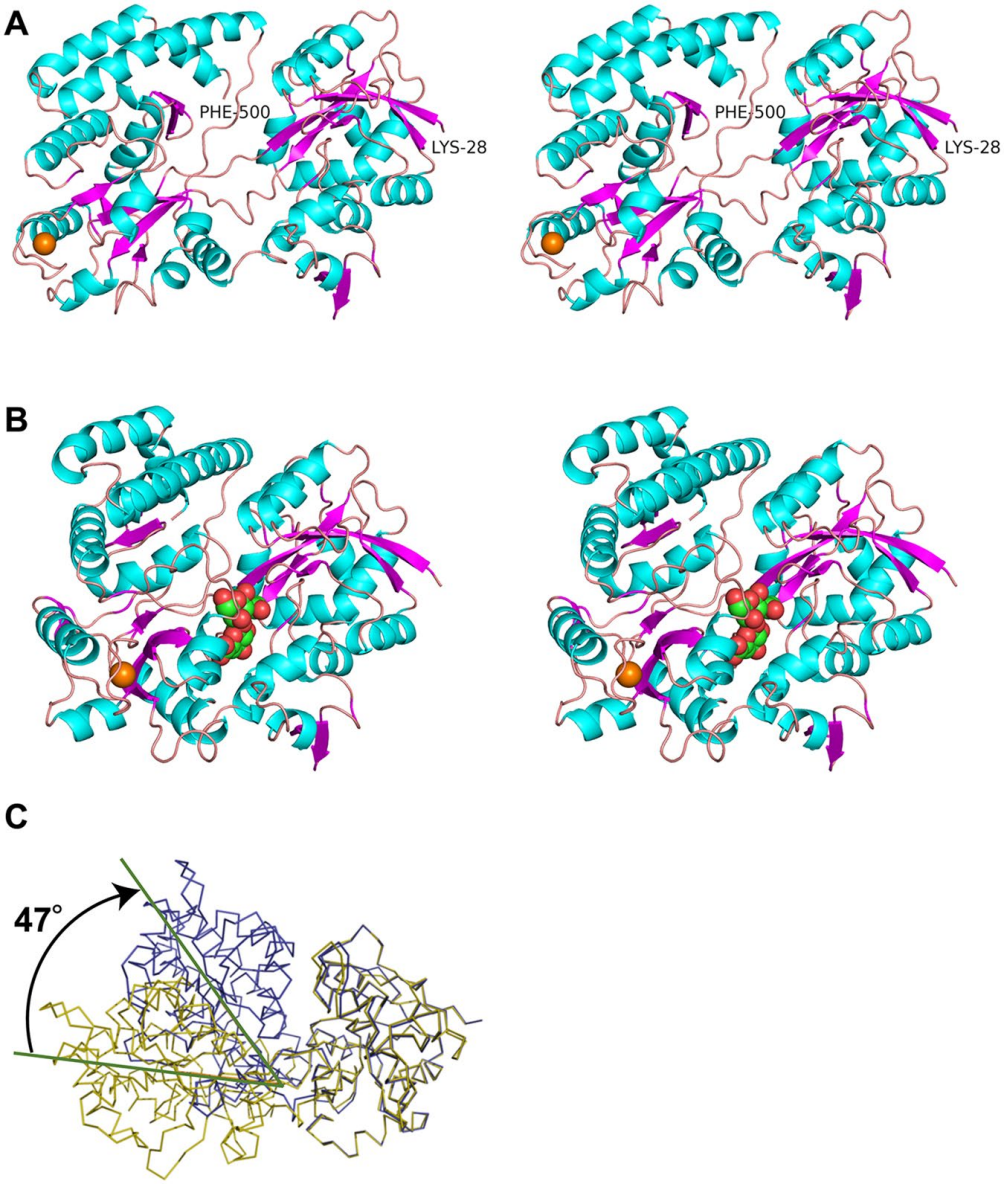
Overall structures of Smon0123. Structures (stereo-diagram with wall-eyed viewing) of ligand-free Smon0123 (N-28/C-5) (**A**) and CΔ0S-bound Smon0123 (N-18/C-5) (**B**). Cyan, α-helices; purple, β-strands; pink, loops and coils. CΔ0S bound to Smon0123 is shown in ball model (green, carbon atom; red, oxygen atom; and blue, nitrogen atom). Orange ball shows the metal ion. Structural comparison with ligand-free Smon0123 (N-28/C-5) (olive) and CΔ0S-bound Smon0123 (N-18/C-5) (blue) (**C**). Both N-domains of ligand-free and -bound Smon0123 proteins were superimposed. There is a structural difference (47° in angle) in the N- and C-domains between C∆0S-free and -bound Smon0123.

The overall structure of Smon0123 (N-18/C-5) in complex with CΔ0S is shown in Fig. 6B. Although Smon0123 (N-18/C-5) consists of 482 amino acid residues, N-terminal 9 amino acid residues (Met1Lys19–Gly26) showed no electron density, possibly due to disorder. Crystal structures of ligand-free Smon0123 (N-28/C-5) (Fig. 6A) and ligand (CΔ4S or CΔ6S)-bound Smon0123 (N-18/C-5) were also determined by molecular replacement with CΔ0S-bound Smon0123 (N-18/C-5). Smon0123 is composed of two major domains (N- and C-domains), and each domain has two small subdomains. N-domain is constituted by residues Pro27–Ile151 (N1 subdomain) and residues Gly328–Ala418 (N2 subdomain), and C-domain contains residues Lys152–Gly327 (C1 subdomain) and Lys419–Phe500 (C2 subdomain). Each domain included a parallel and an antiparallel β-sheet surrounded by many α-helices and formed the α/β sandwich structure. Both N- and C-domains were connected through three loops: residues Tyr146–Ser154 (N1-C1), residues Arg319–Thr324 (C1-N2), and residues Ala414–Ser415 (N2-C2). The ligand CΔ0S was bound to a cleft formed between the two N- and C-domains (Fig. 6B). A metal ion, possibly, a calcium ion based on its coordination, was located in the C-domain, far from the cleft. All the crystal structures of Smon0123 so far determined include metal ion in its molecule, suggesting that the metal ion contributes to protein folding. Because the metal ion is situated far from the substrate-biding site, the metal ion probably has no influence on the substrate-binding ability. Compared with the structure of CΔ0S-bound Smon0123 and that of ligand-free Smon0123, the N- and C-domains of ligand-bound Smon0123 were 47° more closed than those of ligand-free Smon0123 (Fig. 6C). There was no significant difference in the overall structure among Smon0123 proteins in complex with CΔ0S, CΔ4S, and CΔ6S.

### Binding mode of Smon0123 to unsaturated GAG disaccharides

**Figure 7:**
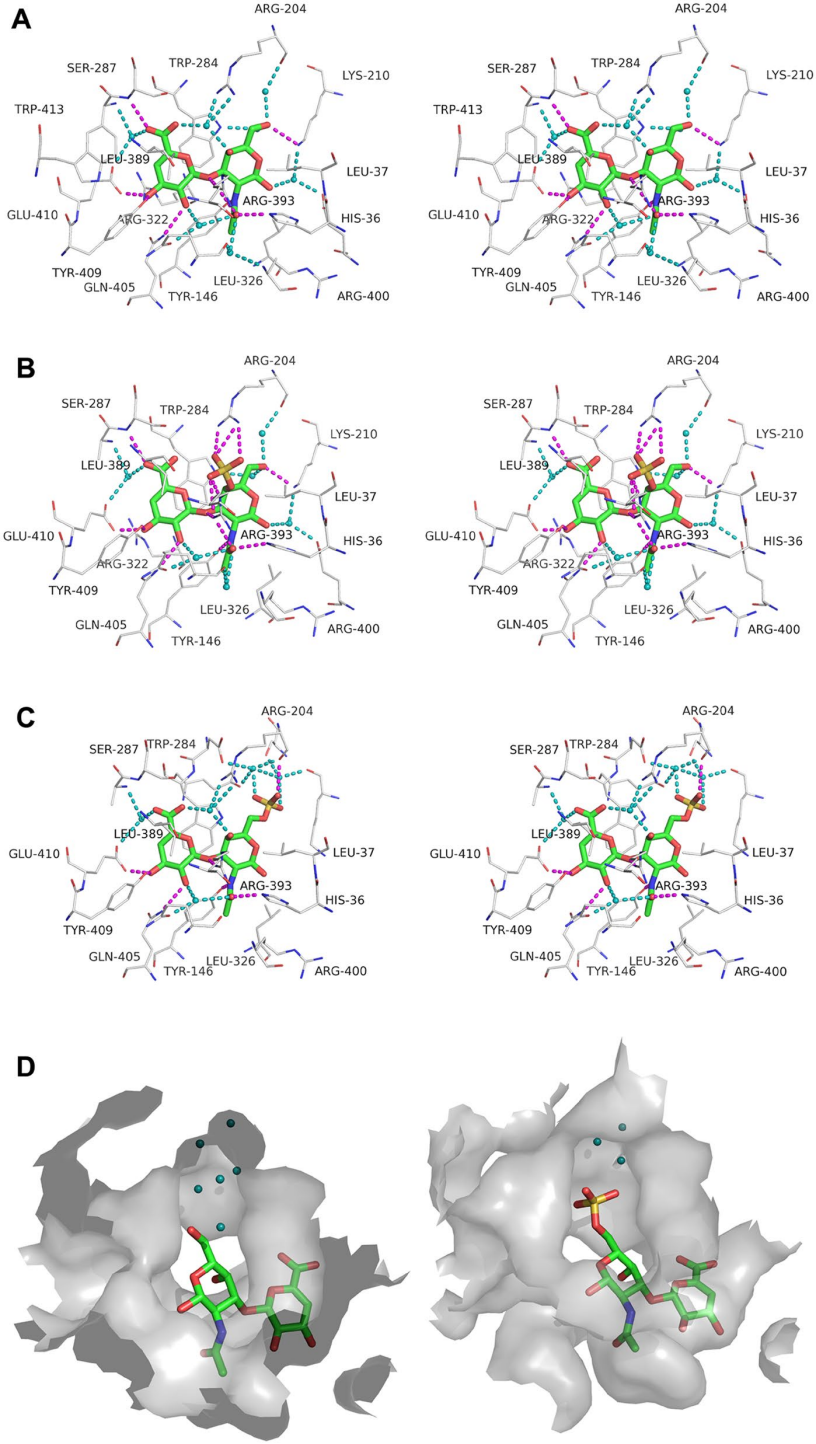
Binding mode of Smon0123 to unsaturated GAG disaccharides. Interactions between Smon0123 and CΔ0S (**A**), CΔ4S (**B**), or CΔ6S (**C**). CΔ0S, CΔ4S, and CΔ6S are shown in sticks (green, carbon atom; red, oxygen atom; blue, nitrogen atom; and yellow, sulfur atom) (stereo-diagram with wall-eyed viewing). Carbon atoms of Smon0123 residues interacting with the substrates are shown in gray lines. Direct and water-mediated hydrogen bonds between Smon0123 and substrates are shown in dashed lines colored with magenta and cyan, respectively. Water molecules (oxygen atoms) are shown as cyan balls. (**D**) Large space for sulfate groups of substrates. In the interaction between Smon0123 and CΔ0S (left), a number of water molecules are located in the space where a sulfate group of CΔ6S is accommodated (right).

Interactions between Smon0123 and each unsaturated chondroitin disaccharide (CΔ0S, CΔ4S, or CΔ6S) are shown in Table 1 and Fig. 7. Unsaturated GAG disaccharides were bound to the cleft of Smon0123 by hydrogen bonds and van der Waals (C-C) contacts. The numbers of hydrogen bonds between Smon0123 and unsaturated chondroitin disaccharides are 9 for CΔ0S (ΔGlcUA, 4; GalNAc, 5), 14 for CΔ4S (ΔGlcUA, 4; GalNAc4S, 10), and 8 for CΔ6S (ΔGlcUA, 4; GalNAc6S, 4). The numbers of C-C contacts between Smon0123 and unsaturated chondroitin disaccharides are 50 for CΔ0S (ΔGlcUA, 27; GalNAc, 23), 48 for CΔ4S (ΔGlcUA, 26; GalNAc4S, 22), and 53 for CΔ6S (ΔGlcUA, 28; GalNAc6S, 25). Smon0123 strictly recognized both residues of unsaturated uronate (ΔGlcUA) and amino sugar (GalNAc/GalNAc4S/GalNAc6S). An aromatic residue, Trp284, showed stacking interactions with both ΔGlcUA and GalNAc/GalNAc4S/GalNAc6S. Numerous water molecules mediated indirect interactions between Smon0123 and unsaturated chondroitin disaccharides by the formation of hydrogen bonds. Basic residues directly or indirectly binding to the sulfate group of CΔ4S (Arg204 and Arg393) and CΔ6S (Arg204 and Lys210) formed a positively charged space for accommodating the sulfate group.

**Table 1:** Interactions between Smon0123 and unsaturated GAG disaccharides

van der Waals (C-C distance <4.5 Å)														
Smon0123 (N-18/C-5)/C∆0S					Smon0123 (N-18/C-5)/C∆4S					Smon0123 (N-18/C-5)/C∆6S				
Sugar	Atom	Protein/Water	Atom	Distance	Sugar	Atom	Protein/Water	Atom	Distance	Sugar	Atom	Protein/Water	Atom	Distance
ΔGlcUA	C1	Trp284	CE2	4.31	ΔGlcUA	C1	Trp284	CD2	4.22	ΔGlcUA	C1	Trp284	CD2	4.06
	C1	Trp284	CE3	3.93		C1	Trp284	CE3	4.00		C1	Trp284	CE3	3.91
	C1	Trp284	CZ2	4.31		C1	Trp284	CE2	4.39		C1	Trp284	CE2	4.29
	C1	Trp284	CZ3	3.89		C1	Trp284	CZ3	3.94		C1	Trp284	CZ3	3.90
	C1	Trp284	CH2	4.10		C1	Trp284	CH2	4.12		C1	Trp284	CH2	4.13
	C1	Trp284	CD2	4.13		C1	Trp284	CZ2	4.36		C1	Trp284	CZ2	4.33
	C3	Trp284	CE3	4.27		C3	Trp284	CE3	4.20		C1	Leu389	CD2	4.21
	C3	Trp284	CZ3	4.14		C3	Trp284	CZ3	4.05		C2	Leu389	CD2	4.09
	C3	Arg322	CZ	4.37		C3	Arg322	CZ	4.38		C3	Trp284	CZ3	4.14
	C3	Glu410	CD2	4.05		C3	Glu410	CD	4.09		C3	Trp284	CE3	4.30
	C4	Trp284	CE3	3.81		C4	Trp284	CE3	3.86		C3	Glu410	CD	4.05
	C4	Trp284	CZ3	4.17		C4	Trp284	CZ3	4.18		C4	Trp284	CE3	3.78
	C4	Leu389	CD2	4.34		C4	Leu389	CD2	4.28		C4	Trp284	CZ3	4.14
	C4	Glu410	CD	3.91		C4	Glu410	CD	3.88		C4	Leu389	CD2	4.13
	C5	Trp284	CE3	3.58		C5	Trp284	CB	4.24		C4	Glu410	CD	3.90
	C5	Trp284	CZ3	4.22		C5	Trp284	CG	4.25		C5	Trp284	CB	4.09
	C5	Trp284	CB	4.14		C5	Trp284	CD2	3.93		C5	Trp284	CG	4.16
	C5	Trp284	CG	4.19		C5	Trp284	CE3	3.58		C5	Trp284	CD2	3.91
	C5	Trp284	CD2	3.91		C5	Trp284	CZ3	4.19		C5	Trp284	CE3	3.60
	C5	Leu389	CD2	3.70		C5	Leu389	CD2	3.71		C5	Trp284	CZ3	4.25
	C6	Trp284	CE3	4.00		C6	Trp284	CB	3.43		C5	Leu389	CD2	3.50
	C6	Trp284	CB	3.36		C6	Trp284	CG	3.87		C6	Trp284	CG	3.88
	C6	Trp284	CG	3.87		C6	Trp284	CD2	4.01		C6	Trp284	CD2	4.10
	C6	Trp284	CD2	4.07		C6	Trp284	CE3	3.91		C6	Trp284	CE3	4.04
	C6	Ser287	CB	3.70		C6	Ser287	CB	3.66		C6	Trp284	CB	3.32
	C6	Leu389	CD2	3.79		C6	Leu389	CD2	3.70		C6	Ser287	CA	4.38
	C6	Trp413	CH2	4.38	GalNAc4S	C1	Leu326	CD2	4.18		C6	Ser287	CB	3.62
GalNAc	C1	His36	CE1	4.29		C2	His36	CE1	4.38		C6	Leu389	CD2	3.69
	C1	Leu326	CD2	4.20		C3	Trp284	CE2	4.10	GalNAc6S	C1	His36	CE1	4.34
	C2	His36	CE1	3.95		C3	Trp284	CH2	4.36		C1	Leu37	CD2	4.39
	C3	Trp284	CE2	4.02		C3	Trp284	CZ2	3.97		C1	Leu326	CD2	4.06
	C3	Trp284	CZ2	3.90		C4	Trp284	CD1	4.39		C2	His36	CE1	4.35
	C3	Trp284	CH2	4.31		C4	Trp284	CE2	4.08		C3	Trp284	CE2	3.97
	C4	Trp284	CE2	3.96		C4	Trp284	CZ2	4.40		C3	Trp284	CZ2	3.89
	C4	Trp284	CZ2	4.31		C5	Trp284	CD1	4.22		C3	Trp284	CH2	4.31
	C4	Trp284	CD1	4.25		C5	Trp284	CE2	3.99		C4	Trp284	CD1	4.06
	C5	Trp284	CE2	4.06		C5	Trp284	CZ2	4.28		C4	Trp284	CD2	4.26
	C5	Trp284	CZ2	4.35		C6	Leu37	CD2	4.22		C4	Trp284	CE2	3.80
	C5	Trp284	CD1	4.25		C6	Arg204	CZ	4.30		C4	Trp284	CZ2	4.20
	C6	Leu37	CD2	4.23		C6	Trp284	CD1	4.24		C5	Trp284	CD1	4.08
	C6	Arg204	CZ	4.24		C7	His36	CE1	4.02		C5	Trp284	CE2	3.92
	C6	Trp284	CD1	4.21		C7	Tyr146	CE1	4.28		C5	Trp284	CZ2	4.25
	C7	His36	CE1	3.95		C7	Tyr146	CZ	4.29		C6	Arg204	CZ	4.34
	C7	Tyr146	CZ	4.35		C7	Leu326	CD2	4.31		C6	Trp284	CD1	3.96
	C7	Tyr146	CE1	4.31		C8	Tyr146	CE1	3.57		C7	His36	CE1	3.88
	C7	Leu326	CD2	4.21		C8	Tyr146	CZ	3.62		C7	Tyr146	CE1	4.32
	C8	Tyr146	CZ	3.76		C8	Leu326	CD2	4.17		C7	Tyr146	CZ	4.33
	C8	Tyr146	CE1	3.70		C8	Arg400	CG	4.20		C7	Leu326	CD2	4.22
	C8	Leu326	CD2	4.00							C8	Tyr146	CE1	3.73
	C8	Arg400	CG	4.05							C8	Tyr146	CZ	3.76
											C8	Leu326	CD2	4.00
											C8	Arg400	CB	4.30
											C8	Arg400	CG	3.93
**Hydrogen bonds (<3.3 Å)**														
**Smon0123 (N-18/C-5)/C∆0S**					**Smon0123 (N-18/C-5)/C∆4S**					**Smon0123 (N-18/C-5)/C∆6S**				
**Sugar**	**Atom**	**Protein/Water**	**Atom**	**Distance**	**Sugar**	**Atom**	**Protein/Water**	**Atom**	**Distance**	**Sugar**	**Atom**	**Protein/Water**	**Atom**	**Distance**
ΔGlcA	O2	Gln405	NE2	2.86	ΔGlcA	O2	Gln405	NE2	2.85	ΔGlcA	O2	Gln405	NE2	2.99
	O3	Tyr409	OH	2.73		O3	Tyr409	OH	2.87		O3	Glu410	OE2	2.68
	O3	Glu410	OE2	2.68		O3	Glu410	OE2	2.69		O3	Tyr409	OH	2.68
	O6A	Ser287	N	2.97		O6A	Ser287	N	2.99		O6A	Ser287	N	2.87
	O2	water80		2.74		O2	water4		2.73		O2	water770		2.74
	O6A	water52		2.74		O6A	water6		2.81		O6A	water757		2.78
	O6B	water143		2.69	GalNAc4S	O1S	Arg393	NE	2.72		O6B	water767		2.55
GalNAc	O3	Arg393	NH2	2.91		O1S	Arg393	NH2	3.00	GalNAc6S	O3	Arg393	NH2	2.96
	O6	Lys210	NZ	2.85		O2S	Arg204	NH1	3.10		O3S	Arg204	O	3.2
	O7	His36	NE2	2.83		O2S	Arg204	NH2	2.83		O7	His36	NE2	2.81
	O7	Arg393	NH2	3.10		O3S	Arg204	NH1	3.17		N	Tyr146	OH	2.86
	N2	Tyr146	OH	2.92		O3	Arg393	NH2	2.91		O1S	water766		3.17
	O1	water72		2.58		O6	Lys210	NZ	2.8		O2S	water795		2.49
	O4	water143		2.69		O7	His36	NE2	2.88		O4	water767		2.67
	O6	water55		2.69		O7	Arg393	NH2	3.08		O7	water770		2.95
	O6	water621		2.57		N	Tyr146	OH	2.88					
	O7	water80		2.94		O1S	water56		2.8					
	O7	water1090		2.98		O6	water301		2.67					
						O6	water324		2.64					
						O7	water4		2.87					
						O7	water543		2.98

### Expression and purification of membrane-bound ABC transporter

Because Smon0123 was identified as an unsaturated GAG disaccharide-binding protein, the cooperative function of Smon0123 and ABC transporter, interacting with each other at the ratio of one to one, should be focused. However, it is difficult to analyze a membrane-bound protein with hetero-subunits. The *S. moniliformis* ABC transporter is considered to consist of tetramer subunits of two membrane-spanning subunits (Smon0121-Smon0122) and ATP-binding subunits (Smon0120-Smon0120) by primary structure-based (homology and topology) analysis. The recombinant ABC transporter (Smon0121-Smon0122/Smon0120-Smon0120) was successfully expressed in *E. coli* cells and purified from the cell membrane to homogeneity by the suitable construction of the expression system of protein subunits and their solubilization with detergents.

Three genes (*smon0120*, *smon0121*, and *smon0122*) coding for Smon0120, Smon0121, and Smon0122, respectively, locate sequentially in that order in the *S. moniliformis* genome and form the operon structure (Fig. 2B). Thus, this operon (*smon0120-0121-0122*) was inserted into an *E. coli* expression vector with a slight modification of 10 histidine residues added at the C-terminus of Smon0122. *E. coli* cell membranes expressing the ABC transporter were obtained from the cell extract by ultracentrifugation and were solubilized with n-dodecyl-β-d-maltoside (DDM). The transporter was purified by a metal affinity chromatography (Ni-NTA) and gel filtration chromatography (HiLoad 16/60 Superdex 200 pg). The sodium dodecyl sulfate (SDS)-polyacrylamide gel electrophoresis (PAGE) profile indicated that the purified sample contained three protein bands corresponding to the transmembrane domain (Smon0121-Smon0122) and ATPase domain (Smon0120) (Fig. 4F). Furthermore, the protein band corresponding to Smon0122 was detected by immunoblotting using anti-histidine tag antibodies (Fig. 4G). The ABC transporter seemed to have been eluted as a tetramer (theoretical molecular mass of approximately 150 kDa) in size on gel filtration (Fig. 4E, red arrow), although the eluted protein was thought to have molecular mass of slightly over 158 kDa based on the elution volume. This difference in molecular mass was possibly due to the presence of a detergent in the eluted buffer. These results clearly demonstrate the tetramer formation of the purified ABC transporter [Smon0121-Smon0122(10xHis)/Smon0120-Smon0120].

### Enhancement of ATPase activity of the ABC transporter by Smon0123 and unsaturated GAG disaccharides

Because ABC transporters generate the energy of ATP hydrolysis to transport substrates^36^, the ATPase activity of the purified Smon0121-Smon0122(10xHis)/Smon0120-Smon0120 was measured to investigate the import of unsaturated GAG disaccharides by the ABC transporter. Although purified Smon0121-Smon0122(10xHis)/Smon0120-Smon0120 in the soluble form with detergents exhibited ATPase activity, there was no significant difference in the activity between the presence and absence of Smon0123 and/or unsaturated GAG disaccharides. Thus, the assay was performed using a proteoliposome^37^.

The purified Smon0121-Smon0122(10xHis)/Smon0120-Smon0120 was reconstructed in liposomes constituted by phospholipids. Various disaccharides were added to the proteoliposome in the presence of Smon0123, and the phosphate ion liberated through ATP hydrolysis was measured by the molybdenum blue method^38^ (Fig. 4H). The ATPase activity in the presence of non-GAG disaccharides as the negative control such as cellobiose and unsaturated alginate disaccharide (d2M) except for *N*,*N*′-diacetylchitobiose was comparable with that in the absence of disaccharides (PLS). The enhancement of the ATPase activity in the presence of *N*,*N*′-diacetylchitobiose was probably due to the similarity in component between GAG disaccharides and *N*,*N*′-diacetylchitobiose. Unsaturated hyaluronan and chondroitin disaccharides (ΔHA, CΔ0S, CΔ4S, and CΔ6S) significantly enhanced the ATPase activity of the proteoliposome, while the enzyme activity in the presence of unsaturated heparin disaccharides (HΔ0S and HΔ6S) was comparable with that in PLS.

## Discussion

In the present study, we found and characterized the bacterial solute-binding protein-dependent ABC transporter for the import system of GAGs for the first time, although a few ABC exporters for GAGs have already been identified in bacteria^39, 40^ and humans^41^. While PTS phosphorylates substrates at the C-6 position during transport, the ABC transporter imports substrates without any substrate modification. The *S. moniliformis* ABC transporter was demonstrated to be active on various unsaturated GAG disaccharides, particularly unsaturated sulfate group-free chondroitin and hyaluronan disaccharides and uniquely (relative to PTS GAG disaccharide import) unsaturated chondroitin disaccharide with a sulfate group at the C-6 position of GalNAc (C∆6S), which is an unsuitable substrate for PTS.

Based on the above-mentioned results and streptococcal GAG assimilation system^12^ (Fig. 1, left), the corresponding *Streptobacillus* model for the degradation and import of GAG was postulated in Fig. 1, right. Because PL family 8 hyaluronate lyase is active on hyaluronan and chondroitin sulfate^42^, extracellular GAGs such as hyaluronan and chondroitin sulfate are depolymerized to unsaturated disaccharides by hyaluronate lyase(s) (Smon0117 and/or Smon0125). Similarly, heparinase (Smon0124) probably acts on extracellular heparin and heparan sulfate. The resultant unsaturated disaccharides are imported to the periplasm through the outer membrane by an unknown channel/transporter. A periplasmic solute-binding protein (Smon0123) captures unsaturated GAG disaccharides with the 1,3-glycoside bond and delivers substrates to an ABC transporter localized in the cytoplasmic membrane. The ABC transporter consists of four subunits (Smon0121-Smon0122/Smon0120-Smon0120) and incorporates unsaturated disaccharides into the cytoplasm with an energy derived through ATP hydrolysis. Because the localization prediction tool *PSORT-B* ^43^ suggests that sulfatase (Smon0126) and UGL (Smon0127) are localized in the cytoplasm, unsaturated disaccharides are degraded to constituent monosaccharides in the cytoplasm by GH 88 hydrolase UGL (Smon0127) before and/or after desulfation by sulfatase (Smon0126). In fact, Smon0127 UGL was found to be expressed in the bacterial cells. No sulfatase activity toward C∆4S was detected in the extracellular fraction (Supplementary Information). The Smon0127 UGL showed a preference for non-sulfated GAG disaccharides, although sulfated GAG disaccharides were also degraded by the enzyme. The substrate specificity of Smon0127 UGL suggests that the sulfatase reaction probably occurs before degradation to constituent monosaccharides by UGL.

Smon0123 was demonstrated to bind to unsaturated hyaluronan and chondroitin disaccharides by DSF and fluorescent spectrum analysis (Fig. 5). The substrate specificity of Smon0123 coincided with that of Smon0121-Smon0122(10xHis)/Smon0120-Smon0120 for ATPase activity (Fig. 4H), indicating that the ABC transporter interacts with substrate-bound Smon0123 (closed form), but not the substrate-free form, and triggers ATP hydrolysis. Distinct from substrate-free Smon0123 in the open conformation, the binding protein in complex with the substrate adopts the closed conformation by accommodating the substrate at the cleft between the N- and C-domains (Fig. 6B). Therefore, the closed conformation of substrate-bound Smon0123 is considered to be important for association with the ABC transporter.

Structure determination of Smon0123 with CΔ6S is particularly important because this result directly demonstrates that CΔ6S, unsuitable for PTS, becomes a substrate for the *S. moniliformis* ABC transporter. The binding mode of Smon0123 to unsaturated chondroitin disaccharides is discussed as follows. The cleft of Smon0123 seems to have spatial allowance. In the interaction between Smon0123 and CΔ0S, numerous water molecules are located in the space where a sulfate group of CΔ4S or CΔ6S is accommodated (Fig. 7D). The sulfate group of each substrate is situated close to the binding site of Smon0123 and directly forms hydrogen bonds with several residues of Smon0123. The C-C contacts of Smon0123 with C∆6S also increase compared with those with CΔ0S. In addition, the pyranose ring of amino sugar in unsaturated GAG disaccharide with 1,4-glycoside bond would be in reverse to that in unsaturated chondroitin disaccharide, suggesting that the low affinity of Smon0123 with unsaturated GAG disaccharides with 1,4-glycoside bond is due to this structural difference in arrangement of the pyranose ring.

Judging from the substrate specificity of Smon0123 (binding protein) and Smon0127 (UGL), the degradation of hyaluronan and chondroitin sulfate by *S. moniliformis* is reasonable (Fig. 3A, 1 and 2, right). Heparin is lethal for this bacterium (Fig. 3A, 3, left), although putative heparinase (Smon0124) is encoded in the GAG genetic cluster (Fig. 2). There is a possibility that heparin oligosaccharide produced from heparin by putative heparinase inhibits bacterial growth. This result suggests that heparin is extremely scarce at the colonization site of *S. moniliformis*, contributing to the compositional analysis of GAGs in extracellular matrices of animal cells. There is a possibility that heparin is rich in the oral cavity in humans but scarce in rodents. Heparin is expected to become a potential anti-*Streptobacillus* agent.

*S. moniliformis* is normally indigenous to rodent oral cavity and belongs to a phylum of fusobacteria. Fusobacteria such as *Fusobacterium* and *Leptotrichia* usually inhabit the oral cavity and gastrointestinal tract of animals including humans^44^. Non-sulfated hyaluronan as well as sulfated GAGs such as chondroitin sulfate are abundant in animal oral cavities^45^. GAGs provide structures of tissues with a strong network due to the high water absorption. Gingiva in the oral mucosa is known to contain particularly rich sulfated GAGs due to the high pressure of mastication^46^. Besides, dermatan sulfate and chondroitin sulfate are rich in connective tissues of the oral mucosa and connective tissue papilla of the anterior and posterior palate in rodents^45^. Therefore, the degradation and import systems of sulfated GAGs in *S. moniliformis* are feasible for its colonization in rodent oral cavities, abundant in sulfated GAGs.

Homologous genes with *S. moniliformis* ABC transporter genes are found in the genome of some fusobacteria. *Fusobacterium mortiferum* (NCBI BioProject ID, 32421) and *Leptotrichia goodfellowii* (NCBI BioProject ID, 43669) form the GAG genetic cluster including ABC transporter genes in bacterial genomes, indicating that the GAG import system is common to these fusobacteria. Some fusobacteria are pathogenic and cause infection in some organs and tissues such as the lung, head neck, cranium, and meninx^47^. This fusobacterial system for the degradation and import of GAG may be involved in colonization and/or infection to host cells, and inhibitors for the bacterial system are expected to develop novel therapy agents.

In conclusion, for the first time, the bacterial import system of sulfated and non-sulfated GAGs was identified as the solute-binding protein-dependent ABC transporter and clarified to be functionally expressed in pathogenic *S. moniliformis* through molecular and structural biology.

## Materials and Methods

### Materials

Unsaturated chondroitin disaccharides (CΔ0S, CΔ4S, and CΔ6S) were purchased from Seikagaku Biobusiness. Unsaturated hyaluronan disaccharide (ΔHA) and unsaturated heparin disaccharides (HΔ6S and HΔNS6S) were purchased from Sigma-Aldrich. A sulfate group-free HΔ0S (heparin disaccharide IV-A) was purchased from Dextra. SYPRO Orange (Invitrogen), DDM (Nacalai tesque), and n-octyl-β-d-glucoside (n-OG) (Dojindo) were commercially available. Oligonucleotides were synthesized by Hokkaido System Science. Restriction endonucleases and DNA-modifying enzymes were purchased from Toyobo. All other analytical grade chemicals used in this study were commercially available.

### Microorganisms, construction of the overexpression system, and protein purification

*Streptobacillus moniliformis* DSM 12112 and its genome were purchased from Deutsche Sammlung von Mikroorganismen und Zellkulturen. Its complete genome sequence is available in GenBank under accession number, CP001779. This bacterium was statically cultured at 37 °C under 5% CO_2_ in 0.8% nutrient broth (0.3% beef extract and 0.5% peptone) (Difco) containing 20% horse serum (Thermo Fisher Scientific) for 24–48 h. To investigate expression of Smon0127 and Smon0123, *S. moniliformis* cells were cultured in nutrient broth and horse serum in the presence or absence of 0.2% GAG [hyaluronic acid (Fluka), chondroitin sulfate C sodium salt (Wako), or heparin sodium salt (Nacalai tesque)] dialyzed against pure water. Detailed experimental procedures for construction of the overexpression system of *S. moniliformis* proteins and protein purification are presented in Supplementary Information.

### Plate method for detection of GAG degradation

Rapid plate method^27^ was adopted to investigate the GAG-degrading ability of *S. moniliformis*. The bacterial cells were cultured in plates containing 1% BSA (Wako), 1% agar, 0.2% dialyzed GAG (hyaluronan, chondroitin sulfate C, or heparin), 0.8% nutrient broth, and 20% horse serum. After addition of 2 M acetic acid (1 ml) to the cultured plates, the remaining GAG was reacted with BSA as white precipitates and clear zones appeared as a halo in GAG-degraded regions.

### Immunoblotting

Antibodies to the purified Smon0123(N-18/C-0) were raised in a rabbit and the serum was used as polyclonal antibodies against Smon0123. To investigate expression of Smon0123 in *S. moniliformis*, the bacterial cells grown in the presence or absence of GAG were subjected to SDS-PAGE followed by immunoblotting using anti-Smon0123 antibodies. Anti-IgG conjugated with horseradish peroxidase (HRP) (GE Healthcare) and Immobilon Western Chemiluminescent HRP substrate (Millipore) were used to detect the target protein. Anti-SpyUGL antibodies^12^ were used as a primary antibody for detection of Smon0127. Furthermore, the purified Smon0121-Smon0122(10xHis)/Smon0120-Smon0120 was subjected to SDS-PAGE followed by immunoblotting using anti-histidine tag antibodies (GE Healthcare).

### Enzyme assay

The Smon0127 UGL was assayed by monitoring the decrease of absorbance at 235 nm derived from the C=C double bond in unsaturated GAG disaccharides. The assay was conducted at 30 °C in reaction mixture of 20 mM Tris-HCl (pH 7.5), 0.2 mM substrate, and the enzyme. Unsaturated GAG disaccharides, ΔHA, CΔ0S, CΔ4S, CΔ6S, HΔ0S, and HΔ6S, were used as substrates. One unit of the enzyme activity was defined as the amount of enzyme required to degrade 1 µmol of substrate per minute as described previously^12, 48^. The enzyme was also assayed using various concentrations (0 to 1 mM) of CΔ0S, and kinetic parameters (*K*_m_ and *k*_cat_) were determined according the Michaelis-Menten equation (Synergy Software).

### DSF

Interactions between Smon0123 and unsaturated GAG disaccharides were investigated by DSF using the MyiQ2 real-time PCR instrument (Bio-Rad). The reaction mixture contained 0 and 1 mM each of disaccharides (CΔ0S and *N*,*N*′-diacetylchitobiose), 50 mM Tris-HCl (pH 7.5), 0.1% SYPRO Orange, and 8.58 µM Smon0123. The temperature was increased from 25 to 95 °C by 0.5 °C per cycle (141 cycles). The RFU values derived from SYPRO Orange hydrophobically bound to unfolded proteins were measured during the heating. The inflection point of the increase in the RFU was determined as melting temperature (*T*_m_). Various disaccharides such as CΔ0S, CΔ4S, CΔ6S, ΔHA, HΔ0S, HΔ6S, and HΔNS6S were used at 50 µM as a ligand.

### Fluorescence spectrum analysis

The fluorescence intensity of Smon0123 with escalated concentration of ligands was measured by spectrofluorometer [FP-6500 (JASCO)]. Parameters under reaction conditions were determined as follows: excitation band width, 1 nm; emission band width, 10 nm; response, 2 s; sensitivity, high; excitation wavelength, 280 nm, start to end emission wavelength, 300 to 500 nm; data pitch, 1 nm; and scan speed, 100 nm/min. The reaction mixture contained 0.1 µM Smon0123, 50 mM Tris-HCl (pH 7.5), and 0 to 20 µM ligands. Various disaccharides such as CΔ0S, CΔ4S, CΔ6S, ΔHA, HΔ0S, HΔ6S, HΔNS6S, *N*,*N*′-diacetylchitobiose, and cellobiose were used as a ligand. The interaction between Smon0123 and GAG polysaccharides such as hyaluronan, chondroitin sulfate C, and heparin was also investigated using BSA as a negative control. The ratio of decreasing fluorescence intensity by addition of increasing ligands in comparison with the intensity in the absence of ligands was plotted and the dissociation constant (*K*_d_) was determined using the least-squares method^49^.

### X-ray crystallography

To determine the three-dimensional structure of Smon0123, the purified protein was crystallized by sitting drop vapor diffusion. The 1 µl of Smon0123 mutants were mixed with equal volume of the reservoir solution for crystallization and a crystal formed at 20 °C for 2 weeks. In the case of ligand-free Smon0123 (N-28/C-5), 21.2 mg/ml Smon0123 (N-28/C-5) was crystallized in the drop consisting of 0.2 M potassium sodium tartrate tetrahydrate, 0.1 M tri-sodium citrate dehydrate (pH 5.6), and 2 M ammonium sulfate. The 12.5 mg/ml Smon0123 (N-18/C-5) was crystallized in the drop containing 1 mM C∆0S, 0.2 M ammonium chloride, 0.1 M MES (pH 6.0), and 20% (w/v) polyethylene glycol (PEG) 6000 for the complex Smon0123 (N-18/C-5)/C∆0S; 1 mM C∆4S, 0.2 M ammonium chloride, 0.1 M HEPES (pH 7.0), and 20% (w/v) PEG 6000 for the complex Smon0123 (N-18/C-5)/C∆4S; and 1 mM C∆6S, 0.2 M potassium thiocyanate, and 20% (w/v) PEG 3350 for the complex Smon0123 (N-18/C-5)/C∆6S. Each single crystal was picked up by a nylon loop, soaked in mother liquor containing 20% ethylene glycol as a cryoprotectant, and frozen in a cold nitrogen gas stream on the beamline BL-38B1 of SPring-8 (Harima, Japan). X-ray diffraction images were collected with a MAR225HE detector (Rayonix) with synchrotron radiation at wavelength 1.00 Å. The data were indexed, integrated, and scaled using the *HKL-2000* program^50^. The structure was determined through molecular replacement method with the *Molrep* program^51^ in the *CCP4* program package. Structure refinement was conducted using the *phenix refine* program in the *PHENIX* program package^52^. After each refinement cycle, the model was adjusted manually with the *winCoot* program^53^. *R*_work_/*R*_free_ values of Smon0123 (N-18/C-5)/C∆0S, Smon0123 (N-18/C-5)/C∆4S, Smon0123 (N-18/C-5)/C∆6S, and Smon0123 (N-28/C-5) are 17.4/20.5, 19.3/21.8, 19.7/24.8, and 20.7/23.6, respectively. The torsion angles of some residues were outlier in the Ramachandran plot analysis due to those distorted β-turn structures and ambiguous electron density. Figures of protein structures were prepared using *PyMOL* ^54^.

### Preparation of proteoliposomes

A soybean l-α-phosphatidylcholine type IV-S (Sigma) was dissolved at 50 mg/ml in 1.5 ml chloroform. Chloroform was removed by rotary evaporation (N-1000-WD, Eyela), and residual solvent was removed under vacuum for 3 h. The resultant dried lipid films were suspended in 20 mM Tris-HCl (pH 8.0) containing 1 mM dithiothreitol (DTT). After sonication 3 to 5 times by probe-type sonicator (Sonics & Materials) on ice, the suspension was frozen in liquid nitrogen and thawed at 37 °C. After 3 repeats of freezing and thawing, this liposome solution was stored at −80 °C. The liposome was thawed at 37 °C and extruded 17 times through a 100 nm polycarbonate membrane by a Mini-Extruder (Avanti Polar Lipids). The 20% n-OG (3.5 µl) was added to the filtered liposome (45 µl) and the mixture was kept at room temperature for 5 min. The 4 mg/ml purified Smon0121-Smon0122(10xHis)/Smon0120-Smon0120 was added to the mixture and kept at room temperature for 10 min. The resultant mixture was diluted to 0.047% of n-OG with 20 mM Tris-HCl (pH 8.0) and placed on ice for 30 min. This solution was used as proteoliposome.

### ATPase assay

The reaction mixture (200 µl) for ATPase assay consisted of proteoliposome (0.1 µM Smon0121-Smon0122(10xHis)/Smon0120-Smon0120), 10 mM MgCl_2_, 2 mM ATP, 20 mM Tris-HCl (pH 8.0), 1.5 µM Smon0123(N-18/C-0), and 0.1 mM each of various disaccharides. The reaction mixture without Smon0123 was incubated at 37 °C and reaction was started when Smon0123 was added. A portion of the reaction mixture (30 µl) was removed every 20 min during 0 to 80 min and added to 30 µl of 12% SDS to stop the reaction. After stopping reaction at 80 min, a mixture of 6% ascorbic acid/1 M HCl and 1% ammonium molybdate (60 µl) was added to the reaction solution (60 µl) and incubated at room temperature for 5 min. Subsequently, a mixture of 2% sodium citrate, 2% sodium metaarsenite, and 2% acetic acid (90 µl) was added to the solution and incubated at 37 °C for 10 min^38^. This reaction mixture was measured in absorbance at 850 nm to determine phosphate ion concentration generated through ATP hydrolysis. ATPase activity was represented as phosphate ion (nmol) produced by 1 mg Smon0121-Smon0122(10xHis)/Smon0120-Smon0120 per 1 min. The value measured using proteoliposome and disaccharides was subtracted from that using liposomes without any proteins and ligands.

## Electronic supplementary material

Supplementary Information
