# Supplementary material for: A bacterial ABC transporter enables import of mammalian host glycosaminoglycans
Source: Sci Rep. 2017 Apr 21;7:1069. doi: 10.1038/s41598-017-00917-y (PMC5430744; doi:10.1038/s41598-017-00917-y)
Supplement: Supplementary file 1 — Supplementary Information [file 41598_2017_917_MOESM1_ESM.pdf]

Supplementary Information

A bacterial ABC transporter enables import of mammalian host glycosaminoglycans

Sayoko Oiki[1], Bunzo Mikami[2], Yukie Maruyama[3], Kousaku Murata[3] & Wataru Hashimoto[1]

[1]Laboratory of Basic and Applied Molecular Biotechnology, Division of Food Science and Biotechnology, Graduate School of Agriculture, Kyoto University, Uji, Kyoto 611-0011, Japan. [2]Laboratory of Applied Structural Biology, Division of Applied Life Sciences, Graduate School of Agriculture, Kyoto University, Uji, Kyoto 611-0011, Japan. [3]Laboratory of Food Microbiology, Department of Life Science, Faculty of Science and Engineering, Setsunan University, Neyagawa, Osaka 572-8508, Japan. Correspondence and requests for materials should be addressed to W. H. (email: whasimot@kais.kyoto-u.ac.jp)

Results

Enzyme proterties of GAG-related enzymes. The substrate specificity of Smon0127 UGL demonstrated that the enzyme preferred sulfate group-free unsaturated GAG disaccharides with the 1,3- rather than the 1,4-glycoside bond, although sulfated substrates (CΔ4S and CΔ6S) also become substrates of Smon0127 UGL. Kinetic parameters toward CΔ0S were determined to be 0.162 mM for K_m and 4.27 /s for k_{cat}. These values are comparable with streptococcal UGL values[1], indicating that Smon0127 UGL physiologically functions in *S. moniliformis* cells.

Regarding sulfatase assay, the spot of CΔ4S on the thin-layer chromatography (TLC)[1] plate in the presence of the extracellular fraction was unchanged, suggesting that sulfatase activity was undetected extracellularly. On the other hand, the substrate was considered to be degraded by intracellular enzymes, such as UGL and/or sulfatase, because the spot of CΔ4S attenuated on the TLC plate.

Crystallization and structure determination of Smon0123. Although Smon0123 with the full-length protein containing a signal peptide was crystallized under some conditions, these crystals were unsuitable for X-ray analysis due to their low-resolution data. Thus, N-terminal and/or C-terminal truncated mutants were constructed in *E. coli* cells, purified to homogeneity, and crystallized. Among mutants, two mutants, N-terminal 18 and C-terminal 5 residue-truncated Smon0123 (N-18/C-5) and N-terminal 28 and C-terminal 5 residue-truncated Smon0123 (N-28/C-5), formed crystals suitable for X-ray crystallography. Smon0123 (N-18/C-5) was crystallized with each unsaturated chondroitin disaccharide (CΔ0S, CΔ4S, or CΔ6S), while Smon0123 (N-28/C-5) was crystallized in the absence of unsaturated GAG disaccharides. Due to heterogeneity of GAG polysaccharides in the polymerization degree and sulfation level, crystallization of Smon0123 with homogeneous GAG disaccharides was attempted in this work. Statistics for data collection and model

refinement are shown in Table S1. Smon0123 (N-18/C-5) crystals with CΔ0S, CΔ4S, and CΔ6S belong to the $P2_12_12_1$ space group, and unit cell dimensions are a=79.7, b=112, c=166 Å; a=80.0, b=113, c=166 Å; and a=80.3, b=112, c=167 Å, respectively. The ligand-free Smon0123 (N-28/C-5) crystal belongs to the $P2_1$ space group with unit cell dimensions of a=73.7, b=142, c=73.7 Å, β=106°. The asymmetry units of ligand-bound crystals are 3 and those of ligand-free crystals are 2. The crystal structure of Smon0123 with CΔ0S was determined at 1.78 Å by molecular replacement with alginate-binding protein (AlgQ1) (PDB ID, 1Y3N) as the initial model.

Methods

Construction of the overexpression system. Overexpression systems for Smon0127, Smon0123, and Smon0121-Smon0122(10xHis)/Smon0120-Smon0120 were constructed in *E. coli* cells as follows. To clone the Smon0127 and Smon0123 genes, polymerase chain reaction (PCR) was conducted in a reaction mixture (10 µl) consisting of 0.2 U of KOD Plus Neo polymerase (Toyobo), 40 ng of the *S. moniliformis* genomic DNA as a template, 0.3 pmol of forward and reverse primers, 2 nmol of dNTPs, 10 nmol of $MgCl_2$, 0.5 µl of dimethyl sulfoxide, and the commercial reaction buffer supplied with KOD Plus Neo polymerase. The oligonucleotides for Smon0127 were 5′-GG<u>CCATGG</u>AATTATTTAAGGAAGTAATAGAAAAAT-3' and 5′-GG<u>CTCGAG</u>CCAATAAATATTCCACTCTGGTTTTAA-3', with *Nco*I and *Xho*I sites (indicated by underlines) added to their 5’ regions, respectively. Those for Smon0123 were 5′-GG<u>CCATGG</u>AAAAATTATTAGCATTAGGACTTTTAG-3' and 5′-GG<u>CTCGAG</u>TTTATTAAAATTCTTGAATGCTTCATT-3', with *Nco*I and *Xho*I sites (indicated by underlines) added to their 5′ regions, respectively. PCR conditions were as follows: 94°C for 2 min followed by 30 cycles of 98°C for 10 s, 35°C for 30 s, and 68°C for

2 min. PCR products were ligated with *Hinc*II-digested pUC119 (Takara Bio), and resultant plasmids were digested with *Nco*I and *Xho*I to isolate the Smon0127 and Smon0123 genes. The correctness of the gene fragments was confirmed by DNA sequencing[2]. DNA fragments for Smon0127 and Smon0123 genes were ligated with *Nco*I and *Xho*I-digested pET21d (Novagen). The pET21d vector was designed to express proteins with a hexahistidine-tagged sequence at the C-terminus. The resultant plasmids for expression of Smon0127 and Smon0123 were designed as pET21d-Smon0127 and pET21d-Smon0123, respectively.

Oligonucleotides for the truncated Smon0123 genes were synthesized by Hokkaido System Science. Forward primers were 5′-AGGAGATATACCATGAAAAAATTATTAGC-3′ (N-0), 5′-AGGAGATATACCATGAAAAAAGAAGAAACTACAACAGGAC-3′ (N-18), 5′-AGGAGATATACCATGGAAACAACAATATTTGCAATGC-3′ (N-28). Reverse primers were 5′-GGTGGTGGTGCTCGATTATTTATTAAAATTCTTGAAT-3′ (C-0), 5′-GGTGGTGGTGCTCGATTAGAATGCTTCATTTTGAATTTGTT-3′ (C-5), 5′-GGTGGTGGTGCTCGATTATTCTAATCTCTTAATATATGCATCCCA-3′ (C-20). Nine mutants were constructed by combining with forward and reverse primers (N-0/C-0, N-0/C-5, N-0/C-20, N-18/C-0, N-18/C-5, N-18/C-20, N-28/C-0, N-28/C-5, and N-28/C-20). The plasmid pET21d-Smon0123 was used as a template. The reaction mixtures for PCR except for the template were prepared as described above. The PCR conditions were as follows: 94°C for 2 min followed by 30 cycles of 98°C for 10 s, 61°C for 30 s, and 68°C for 45 s. The PCR products were digested with *Nco*I and *Xho*I, and cloned with *Nco*I and *Xho*I-digested pET21d by using In-Fusion kit (Clontech). Nucleotide sequences of the truncated genes were confirmed by DNA sequencing. The resultant plasmids for truncated Smon0123 mutants were designated pET21d-Smon0123 [(N-0/C-0), (N-0/C-5), (N-0/C-20), (N-18/C-0), (N-18/C-5), (N-18/C-20), (N-28/C-0), (N-28/C-5), and (N-28/C-20)]. Truncated mutant proteins contained no his-tag sequence.

Overexpression system for Smon0121-Smon0122(10xHis)/Smon0120-Smon0120 was constructed by using the operon gene. To clone the Smon0120-0121-0122 gene fragment with an additional sequence for four histidine residues at C-terminus of Smon0122, oligonucleotides were synthesized by Hokkaido System Science. Forward primer was 5′-AGGAGATATACCATGGCAGAAGTAATATTAAAGAAAGTTG-3′ and reverse primer was 5′-GTGGTGGTGGTGGTGATGATGATGATGTCCTTTTACTGCTCCTAGTGTT-3′. The PCR conditions were as follows: 94°C for 2 min followed by 30 cycles of 98°C for 10 s, 58°C for 30 s, and 68°C for 90 s. Genome of *S. moniliformis* was used as a template. After subcloning in the similar way, the resultant plasmid was designed pET21d-Smon0120-0121-0122. For expression of each protein, *E. coli* host cells were transformed with each of the constructed plasmids. DNA manipulations were conducted as described previously[3].

Protein purification. *E. coli* BL21(DE3)Gold and BL21(DE3)-CodonPlus-RIL (Novagen) were used for expression of Smon0127 and Smon0123, respectively. The *E. coli* cells harboring the expression plasmid (pET21d-Smon0127 or pET21d-Smon0123) were cultured at 30°C in Luria broth[3] (1% tryptone, 0.5% yeast extract, and 1% NaCl) containing 100 µg/ml sodium ampicillin to express the target protein. At 0.3 to 0.7 turbidity at 600 nm, isopropyl-β-D-thiogalactopyranoside (IPTG) was added to the culture at a final concentration of 0.1 mM, and the cells were further cultured at 16°C for 2 days. Cultured cells were collected by centrifugation at 6,700 *g* and 4°C for 10 min, suspended in 20 mM Tris (hydroxymethyl) aminomethane-hydrochloride (Tris-HCl) (pH 7.5).

E. coli BL21(DE3)Gold/pLysS (Novagen) was used as an expression host of Smon0121-Smon0122(10xHis)/Smon0120-Smon0120. The *E. coli* cells harboring the plasmid (pET21d-Smon0120-0121-0122) were cultured at 30°C in Terrific broth[3] (1.2% tryptone, 2.4% yeast extract, 0.4% glycerol, 0.05% $MgSO_4 \cdot H_2O$, 1.4% K_2HPO_4, and 0.26%

KH_2PO_4) containing 1% glucose, 2 mM $MgCl_2$, and 33 µg/ml sodium ampicillin to express Smon0121-Smon0122(10xHis)/Smon0120-Smon0120. At 0.3 to 0.7 turbidity at 600 nm, IPTG was added to the culture at a final concentration of 0.4 mM, and the cells were further cultured at 25°C for 16 h. Cultured cells were collected by centrifugation at 6,700 *g* and 4°C for 10 min, suspended in a standard buffer [20 mM Tris-HCl (pH 8.0), 0.1 M NaCl, 2 mM $MgCl_2$, 10% glycerol, 1 mM phenylmethylsulfonyl fluoride, and 2 mM ethylenediaminetetraacetic acid].

For purification of Smon0127 or Smon0123, the *E. coli* cells containing each target protein were disrupted by ultrasonication (Insonator Model 201M, Kubota) at 0°C and 9 kHz for 10 min, and the supernatant solution obtained by centrifugation at 20,000 *g* and 4°C for 20 min was used as the cell extract. The cell extract was applied to a metal affinity chromatography [TALON (Clontech)]. After being washed with wash buffer [20 mM Tris-HCl (pH 7.5), 0.5 M NaCl, and 10 mM imidazole (pH 8.0)], the protein was eluted with elution buffer [20 mM Tris-HCl (pH 7.5), 0.5 M NaCl, and 0.5 M imidazole (pH 8.0)] and every fraction (3 ml) was collected per 3 min. After being subjected to SDS-PAGE according to the method of Laemmli[4], fractions containing the target protein were combined and dialyzed against 20 mM Tris-HCl (pH 7.5). The protein solution was applied to an anion exchange chromatography [Resource Q (GE Healthcare)] and eluted using linear gradient of NaCl (0 to 1 M) in 20 mM Tris-HCl (pH 7.5). After being subjected to SDS-PAGE, fractions containing the target protein were combined. The protein solution was finally applied to a gel filtration chromatography [HiLoad 16/60 Superdex 200 pg (GE Healthcare)]. After being subjected to SDS-PAGE, fractions containing the target protein were combined and dialyzed against 20 mM Tris-HCl (pH 7.5). The dialysate was concentrated by ultrafiltration [Centriprep (Millipore), molecular mass cut-off, 10 kDa]. The concentrate was used as the purified protein (Smon0127 or Smon0123). Protein concentrations (Smon0127 or Smon0123)

were determined by Bradford method[5] using BSA as a standard protein.

For purification of histidine-tagged sequence-free Smon0123, the cell extract obtained by the method as described above was applied to an anion exchange chromatography [Toyopearl DEAE-650M (Tosoh Bioscience LLC)] using linear gradient of NaCl (0 to 0.5 M) in 20 mM Tris-HCl (pH 7.5). Every fraction (10 ml) was collected per 10 min. The target protein was further purified by HiLoad 16/60 Superdex 200 pg, dialyzed, and concentrated as described above.

For purification of Smon0121-Smon0122(10xHis)/Smon0120-Smon0120, the cell extract obtained as described above was further ultracentrifuged at 100,000 *g* and 4°C for 60 min. The precipitant was suspended in the standard buffer containing 1% DDM [critical micelle concentration (CMC), 0.0087%] and mixed at 4°C thoroughly by gentle rotation for 16 h. The solubilized membrane was subjected to ultracentrifugation at 100,000 *g* and 4°C for 60 min and the resultant supernatant solution was applied to a metal affinity chromatography [Ni-NTA (Qiagen)]. After being washed with the standard buffer containing 20 mM imidazole (pH 8.0) and 0.0087% DDM, the protein was eluted using linear gradient of imidazole (20 to 200 mM) in the standard buffer and every fraction (3 ml) was collected per 3 min. After being subjected to SDS-PAGE, fractions containing the target protein were combined. The protein solution was concentrated by ultrafiltration [Vivaspin 15R (Sartorius), molecular mass cut-off, 30 kDa] and applied to HiLoad 16/60 Superdex 200 pg with the standard buffer containing 0.018% DDM. After being subjected to SDS-PAGE, fractions containing the target protein were combined and concentrated by Vivaspin 15R. The purified transporter concentration was determined using BCA Protein Assay Kit (Thermo Fisher Scientific) using BSA as a standard protein.

Sulfatase assay. The sulfatase activity both in the culture supernatant and toluene-treated cells of *S. moniliformis* was assayed by TLC using CΔ4S as a substrate.

References

1. Nakamichi, Y, Maruyama, Y, Mikami, B, Hashimoto, W & Murata, K. Structural determinants in streptococcal unsaturated glucuronyl hydrolase for recognition of glycosaminoglycan sulfate groups. *J. Biol. Chem.* **286**, 6262-6271 (2011).
2. Sanger, F, Nicklen, S & Coulson, AR. DNA sequencing with chain-terminating inhibitors. *Proc. Natl. Acad. Sci. U. S. A.* **74**, 5463-5467 (1977).
3. Sambrook, J, Fritsh, E & Maniatis, T. Molecular cloning: A laboratory manual 2nd ed Cold Spring Harbor Press New York (1989).
4. Laemmli, UK. Cleavage of structural proteins during the assembly of the head of bacteriophage T4. *Nature* **227**, 680-685 (1970).
5. Bradford, MM. A rapid and sensitive method for the quantitation of microgram quantities of protein utilizing the principle of protein-dye binding. *Anal. Biochem.* **72**, 248-254 (1976).

Table S1. Statistics of Smon0123 for data collection and structure refinement

	**Smon0123 (N-18/C-5) /CΔ0S**	**Smon0123 (N-18/C-5) /CΔ4S**	**Smon0123 (N-18/C-5) /CΔ6S**	**Smon0123 (N-28/C-5)**
**Data collection**				
Space group	$P2_12_12_1$	$P2_12_12_1$	$P2_12_12_1$	$P2_1$
Cell dimensions				
a, b, c (Å)	79.7, 112, 166	80.0, 113, 166	80.3, 112, 167	73.7, 142, 73.7
α, β, γ (°)	90.0, 90.0, 90.0	90.0, 90.0, 90.0	90.0, 90.0, 90.0	90.0, 106, 90.0
Resolution (Å)	50.0-1.78 (1.81-1.78) *	50.0-1.81 (1.84-1.81) *	50.0-2.00 (2.03-2.00) *	50.0-1.78 (1.81-1.78) *
R_{merge}	5.8 (31.0)	11.6 (25.2)	9.4 (40.6)	4.3 (40.7)
I / σ (I)	30.3 (5.15)	37.5 (7.07)	36.3 (7.13)	29.2 (3.00)
Completeness (%)	99.2 (96.9)	98.6 (96.7)	98.3 (95.6)	98.6 (98.6)
Redundancy	3.9 (7.5)	3.7 (7.2)	3.2 (7.4)	2.0 (3.9)
**Refinement**				
Resolution (Å)	41.6-1.78 (1.80-1.78)	39.5-1.81 (1.83-1.81)	45.7-1.99 (2.01-1.99)	39.4-1.78 (1.80-1.78)
No. reflections	143057 (4328)	136214 (4159)	100999 (2530)	138900 (4099)
R_{work} / R_{free}	17.4 (20.6) / 20.5 (28.7)	19.3(22.9) / 21.8 (27.6)	19.7(20.5) / 24.8 (25.9)	20.7 (26.1) / 23.6 (32.1)
No. atoms				
Protein	11525	11573	11499	7701
Sugar / Ca^{2+} / EDO / MES / SO_4^{2-} / TAR	78 / 3 / 16 / 36 / 0 / 0	90 / 3 / 60 / 0 / 0 / 0	90 / 3 / 0 / 0 / 0 / 0	0 / 2 / 32 / 0 / 35 / 10
Water	1153	940	757	769
B-factor (Å^2)				
Protein	23.8	20.1	23.8	23.4
Sugar / Ca^{2+} / EDO / MES / SO_4^{2-} / TAR	10.5 / 16.1 / 20.0 / 23.9 / 0 / 0	12.6 / 18.8 / 26.7 / 0 / 0 / 0	20.7 / 22.0 / 0 / 0 / 0 / 0	0 / 43.1 / 30.8 / 0 / 48.8 / 39.3
Water	24.4	24.1	23.5	29.3
R. m. s. deviations				
Bond lengths (Å)	0.007	0.009	0.02	0.003
Bond Angles (°)	1.07	1.11	1.54	0.635
Ramachandran plot (%)				
Favored region	98.5	97.9	97.5	98.2
Allowed region	1.48	1.89	1.89	1.79
Outlier region	0.07	0.21	0.61	0

*Data for the highest resolution shell is shown in parenthesis.

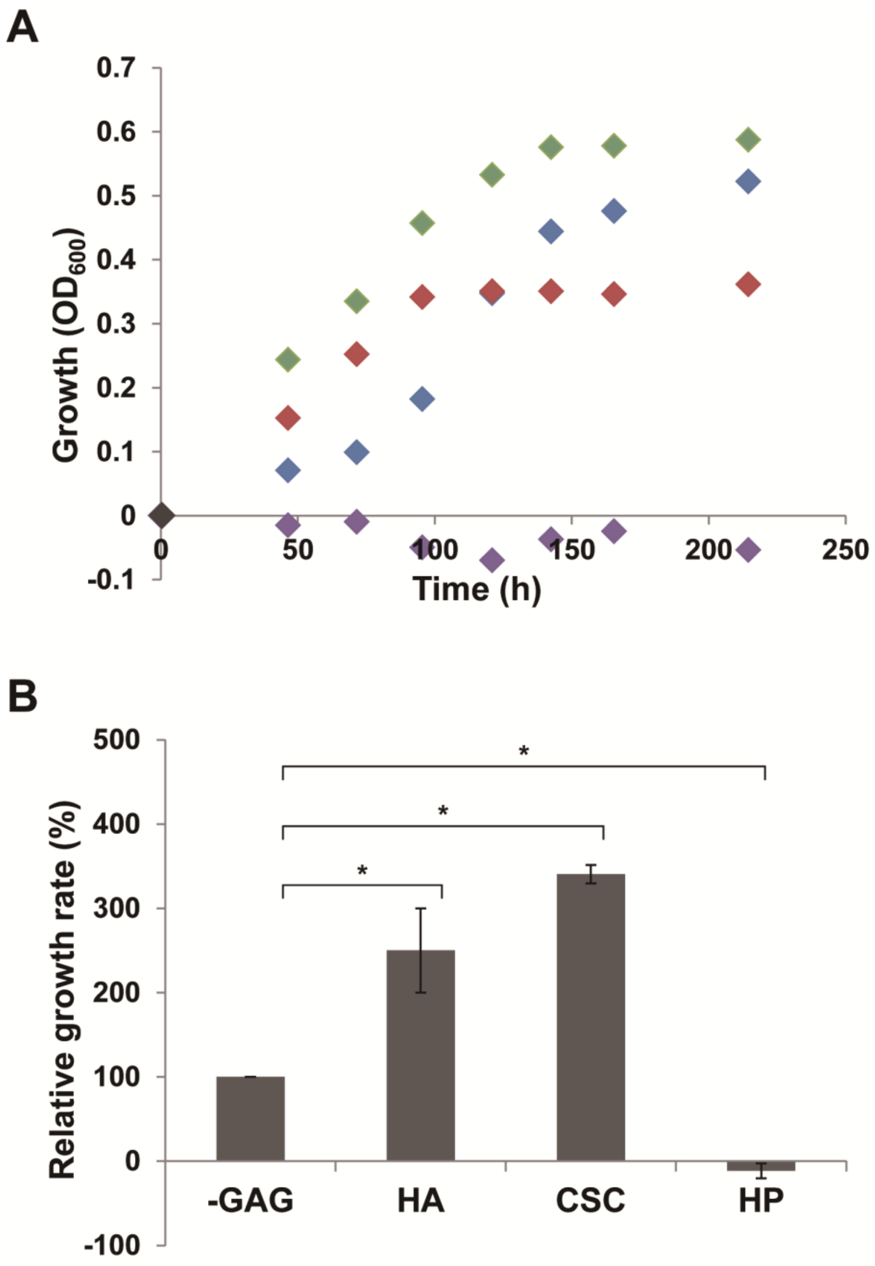

Figure S1. Growth curve of *S. moniliformis*. (**A**) Growth curve of *S. moniliformis* in the presence or absence of GAG. Growth represents optical density at 600 nm (OD_{600}). Blue rhombus, no GAG; red rhombus, hyaluronan; green rhombus, chondroitin sulfate; purple rhombus, heparin. (**B**) Initial relative growth rate in the presence or absence of GAG. –GAG, none of GAG; HA, hyaluronan; CSC, chondroitin sulfate C; HP, heparin. Each data represents the average of triplicate individual experiments (means ± standard deviations). *P<0.05.
